# Engineered liver-derived decellularized extracellular matrix-based three-dimensional tumor constructs for enhanced drug screening efficiency

**DOI:** 10.1093/rb/rbae113

**Published:** 2024-09-06

**Authors:** Shengchang Luo, Qingqing Wang, Miaoting Li, Peiyao Xu, Yicheng Wang, Ying Wang, Ranjith Kumar Kankala, Shibin Wang, Aizheng Chen

**Affiliations:** Institute of Biomaterials and Tissue Engineering, Huaqiao University, Xiamen 361021, PR China; Fujian Provincial Key Laboratory of Biochemical Technology, Huaqiao University, Xiamen 361021, PR China; Institute of Biomaterials and Tissue Engineering, Huaqiao University, Xiamen 361021, PR China; Fujian Provincial Key Laboratory of Biochemical Technology, Huaqiao University, Xiamen 361021, PR China; Institute of Biomaterials and Tissue Engineering, Huaqiao University, Xiamen 361021, PR China; Fujian Provincial Key Laboratory of Biochemical Technology, Huaqiao University, Xiamen 361021, PR China; Institute of Biomaterials and Tissue Engineering, Huaqiao University, Xiamen 361021, PR China; Fujian Provincial Key Laboratory of Biochemical Technology, Huaqiao University, Xiamen 361021, PR China; Institute of Biomaterials and Tissue Engineering, Huaqiao University, Xiamen 361021, PR China; Fujian Provincial Key Laboratory of Biochemical Technology, Huaqiao University, Xiamen 361021, PR China; Institute of Biomedical Engineering, University of Toronto, Toronto, Ontario M5S 3G9, Canada; Toronto General Research Institute, Toronto, Ontario M5G 2C4, Canada; Institute of Biomaterials and Tissue Engineering, Huaqiao University, Xiamen 361021, PR China; Fujian Provincial Key Laboratory of Biochemical Technology, Huaqiao University, Xiamen 361021, PR China; Institute of Biomaterials and Tissue Engineering, Huaqiao University, Xiamen 361021, PR China; Fujian Provincial Key Laboratory of Biochemical Technology, Huaqiao University, Xiamen 361021, PR China; Institute of Biomaterials and Tissue Engineering, Huaqiao University, Xiamen 361021, PR China; Fujian Provincial Key Laboratory of Biochemical Technology, Huaqiao University, Xiamen 361021, PR China

**Keywords:** three-dimensional tumor model, decellularized extracellular matrix, microfluidics, cancer, preclinical drug screening

## Abstract

The decellularized extracellular matrix (dECM) has emerged as an effective medium for replicating the *in vivo*-like conditions of the tumor microenvironment (TME), thus enhancing the screening accuracy of chemotherapeutic agents. However, recent dECM-based tumor models have exhibited challenges such as uncontrollable morphology and diminished cell viability, hindering the precise evaluation of chemotherapeutic efficacy. Herein, we utilized a tailor-made microfluidic approach to encapsulate dECM from porcine liver in highly poly(lactic-*co*-glycolic acid) (PLGA) porous microspheres (dECM-PLGA PMs) to engineer a three-dimensional (3D) tumor model. These dECM-PLGA PMs-based microtumors exhibited significant promotion of hepatoma carcinoma cells (HepG2) proliferation compared to PLGA PMs alone, since the infusion of extracellular matrix (ECM) microfibers and biomolecular constituents within the PMs. Proteomic analysis of the dECM further revealed the potential effects of these bioactive fragments embedded in the PMs. Notably, dECM-PLGA PMs-based microtissues effectively replicated the drug resistance traits of tumors, showing pronounced disparities in half-maximal inhibitory concentration (IC_50_) values, which could correspond with certain aspects of the TME. Collectively, these dECM-PLGA PMs substantially surmounted the prevalent challenges of unregulated microstructure and suboptimal cell viability in conventional 3D tumor models. They also offer a sustainable and scalable platform for drug testing, holding promise for future pharmaceutical evaluations.

## Graphical Abstract

**Figure:**
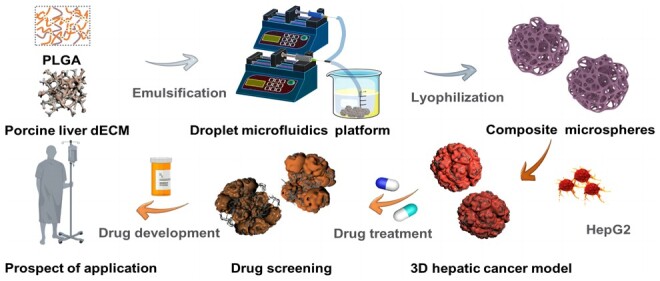

## Introduction

The utilization of *in vitro* three-dimensional (3D) malignant cell culture for anticancer therapeutics addresses the limitations of conventional two-dimensional (2D) cell culture platforms and the associated high costs and ethical concerns of xenograft animal models [1, 2]. Biomimetic scaffold-based 3D tumor models have emerged as promising disease models, enabling the faithful replication of the intricate microenvironment presenting natural 3D malignant tumors [3]. Moreover, these models offer customization using various biomaterials, such as gelatin methacryloyl (GelMA) and poly(lactic-*co*-glycolic acid) (PLGA), rendering them versatile tools for preclinical drug screening [4, 5]. However, a significant challenge in scaffold-based 3D tumor models is the absence of a preexisting extracellular matrix (ECM), which is crucial for facilitating cell adhesion and promoting proliferation. The lack of ECM can result in inadequate cell–matrix interactions, thus failing to replicate the physical and biochemical cues of the native tumor microenvironment (TME) [6, 7]. As a result, these biomimetic scaffolds have limited potential to replicate the hallmark features of the native TME accurately.

The presence of organotypic ECM holds paramount significance, as intact native ECM, provides a platform for tumor growth, differentiation and metastasis, while also contributing to the development of long-term tumor resistance [8, 9]. Recent advancements have facilitated the development of tissue-mimetic scaffolds, enabling the efficient construction of *in vitro* 3D tumor models [10, 11]. In these models, tumor cells are cultured with the scaffolds, resulting in the deposition of pathological ECM components onto the scaffolds in a culture-dependent manner. This process mimics the structural and biochemical characteristics of *in vivo* tumor tissue, which significantly enhanced the advances in tumor research. However, the organ/tissue-derived ECM composition seen in natural tumors was not fully replicated through these approaches, requiring an improvement of *de novo* ECM levels [12]. Unfortunately, achieving a higher abundance of organotypic ECM is often challenging due to significant variations associated with culture-dependent scaffolds. Nevertheless, it should be noted that the ECM obtained from 2D monolayer cultures has been shown to partially represent the phenotypes and profiles of malignant tumors *in vitro*, this method falls short in replicating tissue-specific ECM, which plays a pivotal role in signaling cues compared to complex ECM-mimetic scaffolds [3, 6]. To address these limitations, utilizing decellularized ECM (dECM) allows the retention of bioactive components that are crucial for mimicking the organ-specific tumor environment, thereby enhancing the biomimetic property of our models and improving the efficacy and relevance of drug screening assays. This approach can potentially enhance the performance of drug screening assays by better mimicking the natural ECM found in specific tissues [13].

In an increasing number of studies, researchers have turned to dECM as a biomaterial to establish tissue-specific bioactive platforms, aiming to retain the bioactive composition of the organotypic ECM to the greatest extent possible [14–16]. Although cancer models based on dECM are relatively few, several approaches have been explored, including enzymatic processing and direct use of digested dECM or its incorporation into composite hydrogels. For instance, liver-derived dECM has been enzymatically digested to create thermosensitive hydrogels, combined with other hydrogels such as GelMA or hyaluronic acid [17–19]. The manufacture of such composite methods could enhance the biomimetic nature of malignant tumors, as well as achieve the tunability of dECM-based biomaterials in the field of drug screening [11]. Despite the outstanding features of dECM-based hydrogels, such as tunable water retention and mechanical properties, these are the requirements of preclinical drug screening that demand consistent reproducibility among scaffolds. The direct mixing of dECM with various hydrogels could also result in the rapid loss of dECM components [14]. Compared to the unpredictable morphology seen in dECM-based hydrogels, achieving uniform organotypic tumor morphology *in vitro* during the preclinical testing stages is of utmost importance [20].

As a proof of concept, a unique method based on the encapsulation of dECM derived from porcine liver within highly porous microspheres (dECM-PLGA PMs) was developed in this article, utilizing a specialized droplet microfluidic technique. This innovative approach facilitated the *in vitro* development of 3D liver cancer models, integrating crucial components, such as hepatic tumor cells (HepG2), and liver-derived dECM, ensuring elevated levels of cell viability [21, 22]. Particularly, we have meticulously optimized the preparation process of these composite PMs, ensuring that the dECM-enriched PLGA PMs could be utilized to construct *in vitro* monotypic tumor models effectively. Furthermore, we have conducted a comprehensive proteomic analysis to assess the impact of leveraging porcine liver-derived dECM on HepG2. The generated dECM-PLGA PMs-based microtumors successfully replicated the hallmark of anticancer drug resistance observed in solid tumors. Importantly, they exhibited a significant difference in half-maximal inhibitory concentration (IC_50_) compared to traditional 2D monolayer cultures. This difference underscored the importance of the porcine-derived ECM in influencing drug sensitivity to two chemotherapeutic drugs, sorafenib (SOR) and cisplatin (CIS). To emphasize the specific focus of our study on the distinctive benefits of 3D microenvironments, we compared traditional 2D monolayer cultures directly with 3D microsphere models, highlighting the unique interactions and drug responses facilitated by the 3D architecture and dECM integration. Overall, dECM-PLGA PMs-based 3D cancer microtumors simulated the core hallmarks of liver cancer, exhibiting a valuable tool that could be applied to screen anticancer candidates in the future.

## Materials and methods

### Materials

High glucose-Dulbecco's modified eagle medium (HG-DMEM), fetal bovine serum (FBS), penicillin–streptomycin (P–S), ethylenediaminetetraacetic acid–trypsin and phosphate-buffered saline (PBS, pH 7.2–7.4) were purchased from Biological Industries (Kibbutz Beit-Haemek, Israel). PLGA (66–107 kDa, lactide:glycolide 75:25), poly(vinyl alcohol) (PVA), gelatin (porcine skin, type A), dichloromethane (DCM) and porcine-derived pepsin were purchased from Sigma-Aldrich Co., Ltd (St Louis, CA, USA). Paraformaldehyde (PFA, 4%), 6-diamino-2-phenylindole (DAPI), propidium iodide (PI), live dead kit cell imaging kit, tetramethyl rhodamine isothiocyanate (TRITC)-phalloidin, sodium dodecyl sulfate (SDS), triton-X-100, bovine serum albumin (BSA) protein assay, bicinchoninic acid (BCA), cell counting kit (CCK)-8 and acridine orange/ethidium bromide (AO/EB) dual stain kit were procured from Solarbio Science & Technology Co., Ltd (Beijing, China). The sulfated glycosaminoglycan (GAG) and porcine total collagen assay kits were obtained from Shanghai FANKEL Industrial Co., Ltd (Shanghai, China). CIS, doxorubicin (DOX) and SOR were purchased from Aladdin Biochemical Technology Co., Ltd (Shanghai, China). Hematoxylin and eosin (H&E) staining reagents were obtained from Solarbio Science & Technology Co., Ltd. The permeable working solution (P0097) was obtained from Beyotime Biotechnology (Shanghai, China). No further purifications were executed for all chemicals.

### Methods

#### Preparation of decellularized porcine liver

The porcine liver was sliced into thin sections ranging from 2 to 5 mm in thickness. The subsequent process involved preparing the liver-derived dECM in a beaker at 500 rpm and included several steps: initially, the liver tissue was washed with PBS three times for 10 min each, followed by treatment with 1% SDS for 48 h. PBS was used to wash the tissue to eliminate any residual SDS, and this step was repeated three times for 2 days. The final decellularized tissue was then collected and subjected to freeze-drying at −80°C.

#### Evaluation of the liver-derived dECM

The microstructures of both the native liver tissue and the liver-derived dECM were analysed using field scanning electron microscopy (SEM) from Hitachi (Tokyo, Japan), with the acceleration voltage set to 5 kV. Moreover, samples of either native or liver-derived dECM were soaked in 4% PFA, followed by stained with H&E and the photos were captured using an optical microscope (ECLIPSE Ei, Nikon, Japan). In addition, the nuclei in these samples were counter-stained using DAPI, a standard procedure for both native and liver-derived dECM. Finally, the images of these stained samples were captured using confocal laser scanning microscope (CLSM, TCS SP8 system from Leica Microsystems, Wetzlar, Germany).

#### Preparation and evaluation of liver-derived dECM

Following the decellularization process, the liver-derived dECM was cut into smaller pieces of approximately 5 mm^2^ each. Subsequently, 1 g of the liver-derived dECM pieces was enzymatically digested using a 2 mg/ml porcine pepsin solution in 0.01 M hydrochloric acid. The digestion was conducted at 90 rpm for 48 h at room temperature to ensure a thorough breakdown of the ECM components into a soluble form. Then, the mixture was centrifuged at 3000 rpm for 5 min to facilitate the removal of any undissolved materials and ensure the purity of the soluble components. The supernatant containing the soluble dECM components was carefully decanted to avoid disturbing the pellet. The pH of the supernatant was then adjusted to a physiological range of 7.2–7.4 using 0.1 M sodium hydroxide to neutralize the acidity from the pepsin digestion.

The neutralized solution was subsequently lyophilized to obtain a dry powder form of the liver-derived dECM. The dry powder could ensure the stability and ease of storage of the ECM components, which could be readily reconstituted in an appropriate buffer for various downstream experiments.

The liver-derived dECM were subjected to protein extraction using the sodium dodecyl sulfate-Tris lysis method and quantified by the BCA assay. Post-sample preparation, the samples were lyophilized and reconstituted in 40 µl trypsin buffer, followed by incubation at 37°C for 16–18 h. For liquid chromatography, solution A was 0.1% formic acid in the water, and solution B was 0.1% formic acid in 84% acetonitrile–water. The liquid chromatography column (0.15 mm × 150 mm, RP-C18, Column Technology Inc., USA) was equilibrated with 95% solution A. Samples were loaded by an autosampler onto Zorbax 300SB-C18 peptide traps (Agilent Technologies, Wilmington, DE, USA), followed by separation on the liquid chromatography column. The liquid chromatography gradient was set as follows: 0–110 min, a linear gradient of solution B from 4% to 50%; 110–114 min, a linear gradient from 50% to 100%; 114–120 min, and solution B maintained at 100%. The digested peptides were separated by capillary high-performance liquid chromatography and analysed using a Q Exactive mass spectrometer (Thermo Fisher, Waltham, MA, USA). The analysis time was 120 min with positive ion detection mode. The mass-to-charge ratios of peptides and their fragments were collected by acquiring 10 MS2 scans for every full scan. The raw files from mass spectrometry were processed using MaxQuant 1.5.5.1 software (Max Planck Institute of Biochemistry, Martinsried, Germany) for database searching, resulting in protein identification and quantification.

#### Fabrication of dECM-PLGA PMs *via* microfluidic technology

PLGA PMs were fabricated compartmentally based on our previously reported protocol [22, 23]. Briefly, the emulsified oil droplets, enveloped with a gelatin aqueous phase, were introduced into the continuous flowing PVA aqueous solution through a coaxial nozzle in the customized droplet microfluidic setup. Subsequently, the microspheres underwent solvent extraction and lyophilization procedures, resulting in the formation of PLGA PMs. For the fabrication of dECM-PLGA, the dECM was dissolved into the gelatin aqueous solution, which was enveloped by the PLGA during the emulsion process.

#### Optimization and characterizations of dECM-PLGA PMs

The SEM images of samples were obtained after being sputter-coated with gold for 1 min. Additionally, the particle size and pore size of PMs were measured with the representative results by SEM equipment during data collection. These size distributions of PMs were analysed with Image J software (National Institutes of Health, Bethesda, MD, USA). The full-factorial experimental design based on Minitab software (Minitab, LLC, State College, PA, USA) was plotted to explore the effect of parameters including dECM concentration, gelatin concentration and ultrasonic power on the morphology of PMs. The degradation of microspheres occurred within a buffer designed to replicate the pH and temperature conditions of the human physiological environment. In brief, 20 mg of sterile PMs were introduced into 10 ml PBS buffer for one week in the rotary incubator (37°C, 100 rpm), and the pH of PBS (0–3 weeks) was also evaluated. The resultant PMs on days 1 and 7 were lyophilized and representative SEM images were captured to visualize the structure changes. Fourier transform infrared (FTIR, Nicolet iS50, ThermoFisher, Waltham, USA) spectra of raw PLGA, dECM, PLGA PMs and dECM-PLGA PMs were recorded amid a wavenumber range of 4000–400 cm^−1^. The atomic chemical composition of the scaffolds was analysed using X-ray photoelectron spectroscopy (XPS, Thermo Kalpha, Thermo, LD, UK). Further, the dECM-PLGA PMs were fixed with 4% PFA and stained with Collagen type I (Col-I) to explore the occurrence of dECM in the composite PMs.

#### dECM loading rate in the dECM-PLGA PMs

The optimized dECM-PLGA PMs were dissolved in the DCM to remove the polymer component, while the undissolved remnant of dECM was sucked out carefully with a pipette and subsequently dried in the glass dish to weigh. The dECM loading rate (*R*) could be calculated as follows:

$$R\left(\%\right)=M_{1}/M_{2}\times 100$$

where the *M*_1_ is the dECM loaded in the composited PMs, *M*_2_ is the weight of dECM-PLGA PMs.

#### Cell culture

HepG2 and mouse fibroblast cell line (L929) were obtained from a commercial source (Procell, Wuhan, China), and the HG-DMEM containing 10% FBS and 1% P–S in an incubator at 37 °C under 5% CO_2_ with saturated humidity.

For PMs-based cell culture, HepG2 was incubated with the PMs in a 50 ml sterile centrifuge tube (5 × 10^4^, 5 ml) under dynamic culture for cellular adhesion to the scaffolds. Additionally, a thermostatic aseptic shaker (37°C, 90 rpm) was used for placing the sealed centrifuge tube, where the culture media were changed every 2 days.

#### Cell compatibility of the dECM-PLGA PMs

The aseptic dECM-PLGA PMs were incubated in the HG-DMEM to prepare the leach liquor (72 h, 37°C). The cytotoxicity of the dECM-PLGA PMs and PLGA PMs (0.5, 1 and 2 mg/ml) to L929 was analysed at 450 nm using CCK-8 assay, while the HG-DMEM was employed as controls. The cell viability was calculated as below:

$$\begin{array}{c} \text{Cell}\;\text{viability}\;\left(\%\right)=\left(\text{sample}\;\text{group}\;{\text{OD}}_{450\;\text{nm}}-\text{blank}\;\text{group}\;{\text{OD}}_{450\;\text{nm}}\right)/\\ \left(\text{positive}\;\text{control}\;\text{group}\;{\text{OD}}_{450\;\text{nm}}-\text{blank}\;\text{group}\;{\text{OD}}_{450\;\text{nm}}\right)\times 100 \end{array}$$

Correspondingly, the live and dead staining of L929 cells was also conducted at the concentration of 2 mg/ml leach liquor and captured the images by fluorescence microscope (Axio Observer A1, ZEISS, Oberkochen, Germany).

#### Proliferation activity assay of HepG2

The CCK-8 assay of HepG2 loaded in the PMs at 1, 3, 5 and 7 d was determined to quantify the cell proliferation capacity. Meanwhile, the bright-field of cell-laden PMs was also captured at 1, 3, 5 and 7 d with an optical microscope (Inverted microscope, ECLOPSE Ts2, Nikon, Tokyo, Japan). Further, nuclear DNA was dyed with DAPI on corresponding days, and the representative results were captured with CLSM to illustrate the cellular distribution in scaffolds.

The cell cycle of HepG2 on PMs was also detected by flow cytometry (FCM, BD Melody, BD Biosciences, NY, USA). Briefly, HepG2 was enzymatically collected from respective groups to make a single-cell suspension and centrifuged at 1200 rpm for 4 min. Then, the resultant cells were washed with PBS and fixed with 70% ethanol overnight at 4°C. Further, PI and Ribonuclease A were added into a centrifuge tube. Lastly, the G1, G2 and S-phase proportions were analysed by FCM and cell cycle phase mode in Flow Jo software (LLC, Ashland, OR, USA).

The cell-loaded PMs at 3 days were stained with Ki-67 to compare cell proliferation influenced by dECM after 3 d incubation. Ki-67 is a protein marker for cell proliferation, particularly in cancer. High Ki-67 levels indicate rapid cell division and potentially more aggressive tumors [24]. It is essential for evaluating cancer severity and guiding treatment strategies, typically identified through immunofluorescence assay.

Briefly, the cell-loaded PMs were fixed with pure methanol for 10 min. Subsequently, a permeable working solution was added for 5 min and blocked with normal goat serum/BSA/glycine in PBS-Tween for 2 h. Then, the cells were incubated with an anti-Ki-67 antibody (ab15580, Abcam, Shanghai) overnight at 4°C. Cells were labeled with a secondary antibody. Images were captured using CLSM against the counter-stained nuclear DNA with DAPI. The representative results illustrated the proliferative activity of cells on the PMs.

#### Construction of in vitro 3D hepatic tumor model

To establish *in vitro* the 3D hepatic tumor model, HepG2 cells were incubated with dECM-PLGA PMs to construct the microtumors, where the cell-laden PLGA PMs were set as a control group. Live and dead assay on 1 d and 7 d was conducted, and the survival percentage was calculated as follows:

$$\text{Survival}\;\text{percentage}\;(\%)=I_{\text{green}}/(I_{\text{green}}+I_{\text{red}})\times 100$$

where *I*_green_ and *I*_red_ represent the fluorescence intensities per field of green and red fluorescence, respectively.

Cytoplasmic actin microfilament structures were also located with TRITC-phalloidin according to the manufacturer's protocol. The critical protein, Multidrug resistance protein 2 (MRP-2), in the section of cell-laden PMs on day 7 was subjected to immunoassay to visualize the expression levels inside the microtissues. The representative samples were imaged by CLSM. Additionally, the samples on day 7 were fixed with 4% PFA, and the sections were dyed with H&E dual stain to explore the cellular infiltration attributes.

Further, the MRP-2, *β*-catenin and *p*-*β*-catenin levels of cell-laden scaffolds were, respectively, determined using a Western blotting assay. The qualitative results of the protein expression levels could be analysed using Image J software. The *β*-actin served as a loading control to normalize the expression levels of the proteins of interest, ensuring that variations in protein expression were not due to differences in the amount of total protein loaded on the scaffolds.

#### Chemotherapeutic drug cytotoxicity test

To evaluate the drug sensitivity of the *in vitro* 3D hepatic tumor model to anticancer drugs, HepG2 cells (5 × 10^4^ cells per well) were incubated with PMs as testing units (24-well plates, five cell-laden PMs and 5 × 10^4^ cells per well) for drug screening. After 3 d dynamic culture, cell-laden PMs were incubated with CIS and SOR at the concentration of 0.01, 0.1, 1, 10, 100 μM for 48 h, and HG-DMEM, HG-DMEM with cell-laden PMs served as control. Meanwhile, 2D monolayer tumor models were also employed. About 5 × 10^4^ HepG2 cells per well were inoculated into 24-well plates and subsequently treated with the same concentrations in 3D culture. After 48 h of incubation, the CCK-8 working solution was added in an amount of one-tenth of the volume of the culture solution. The absorbance values at 450 nm for each treatment group were assessed by washing off the supernatant after 1 h of incubation, and the corresponding cell viability was eventually calculated.

#### Features of in vitro 3D hepatic tumor model

AO/EB dual staining was successfully used to assess cell viability in cell-laden PMs. Herein, the cell-laden PMs were stained after treatment with antitumor drugs at a concentration of 100 μM, respectively, according to the manufacturer’s instructions. DOX, as a red-fluorescent drug, was used to visualize the capacity of the cellular internalization ability of cell-laden PMs at time points of 4 and 12 h, and nucleas was counter-stained with DAPI. The representative models were observed using CLSM.

#### Statistics

All the data were analysed and organized using GraphPad Prism 8.0.1 software (San Diego, CA, USA), and the image processing was performed with Image J software. The corresponding data analysis was solved based on recommended approaches in these software tools to determine the comparison of the variances of data as *P < 0.05 (statistically significant), **P < 0.01 (moderately significant), ***P < 0.001 (highly significant) and ****P < 0.0001 (extremely significant).

## Results and discussion

### Decellularization and characterization of porcine liver-derived dECM

**Figure 1.**
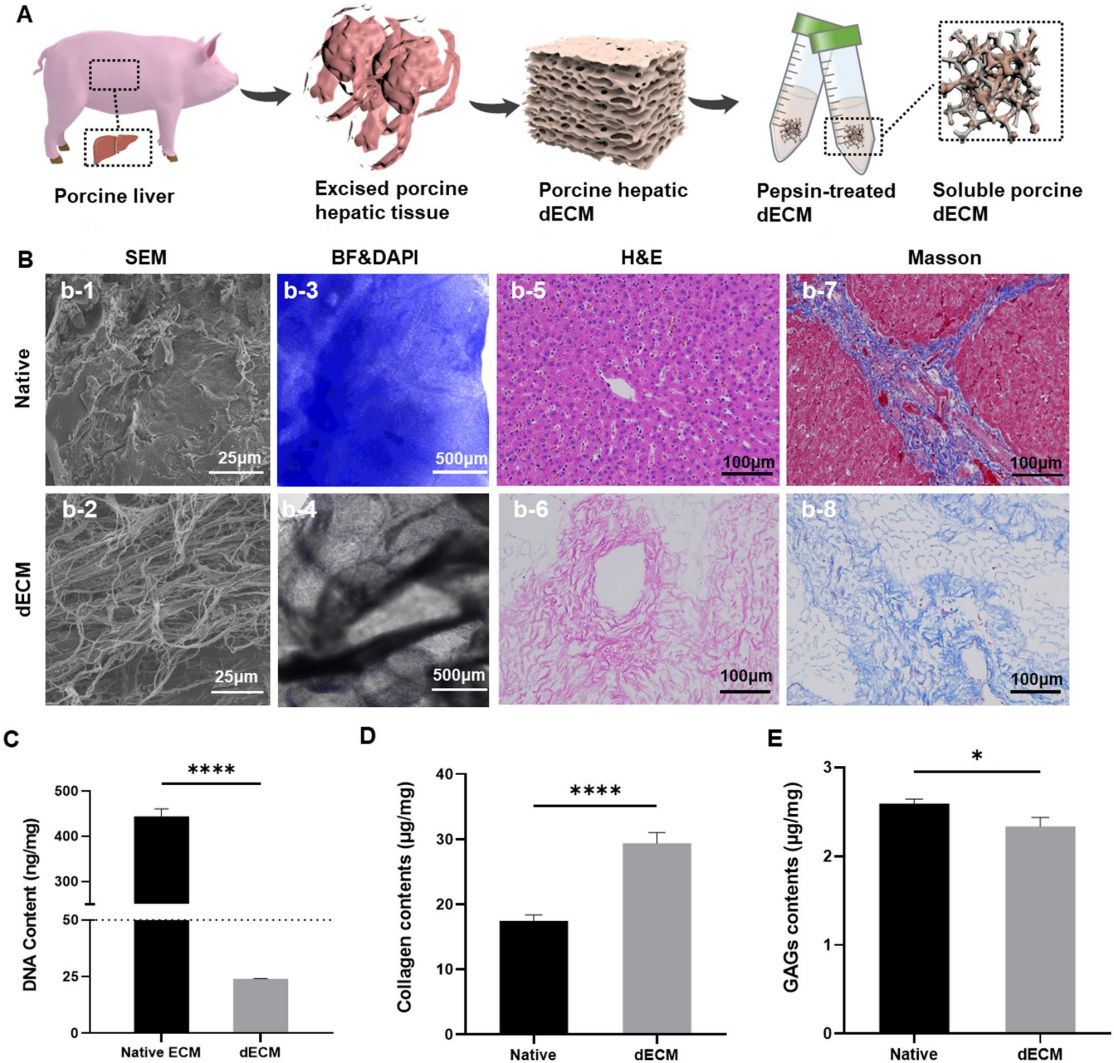
Decellularized and characterization of porcine liver. (A) Schematic overview of the decellularization and pepsin-treated dECM process. (B) Morphological overview of liver-derived dECM, including SEM micrographs of the native liver ECM (b-1) and porcine liver-derived dECM (b-2). Scale bar = 25 μm. Fluorescence imaging of DNA content in the native liver ECM (b-3) and porcine liver-derived dECM (b-4). Scale bar = 100 μm. H&E staining and Masson staining of native liver ECM (b-5 and b-7) and porcine liver-derived dECM (b-6 and b-8). Scale bar = 100 μm. (C) Quantitative analysis of DNA content of the native liver ECM and porcine liver-derived dECM. ****P <* *0.0001. (D) Analysis of collagen-I content in native liver ECM and porcine liver-derived dECM. ****P <* *0.0001. (E) Analysis of GAGs content in native liver ECM and porcine liver-derived dECM. *P <* *0.05.

**Figure 2.**
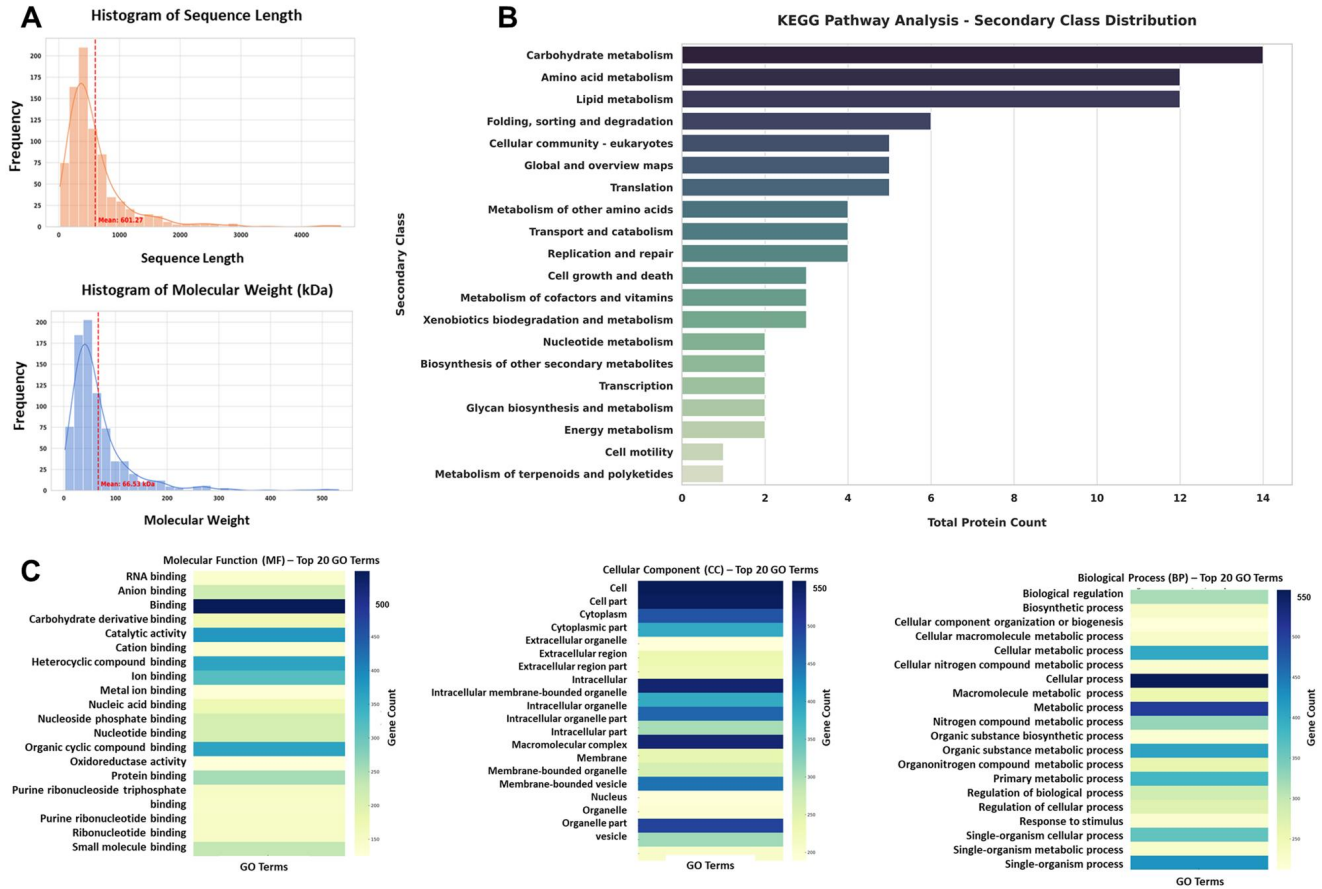
Composition of the liver-derived ECM. (A) Molecular weight distribution and sequence distribution length based on compositions in the liver-derived dECM. (B) KEGG analysis of porcine liver-derived dECM, the secondary class, was used to stand the relative to key pathways relations of porcine liver-derived dECM. (C) GO analysis of protein composition (CC), biological processes (BP) and involvement of the dECM proteins in different molecular functions (MF). The top 10 GO terms were used to introduce the main functions of dECM. Statistical significance was determined at a defined level of *P *<* *0.05 for all proteomic analyses.

**Figure 3.**
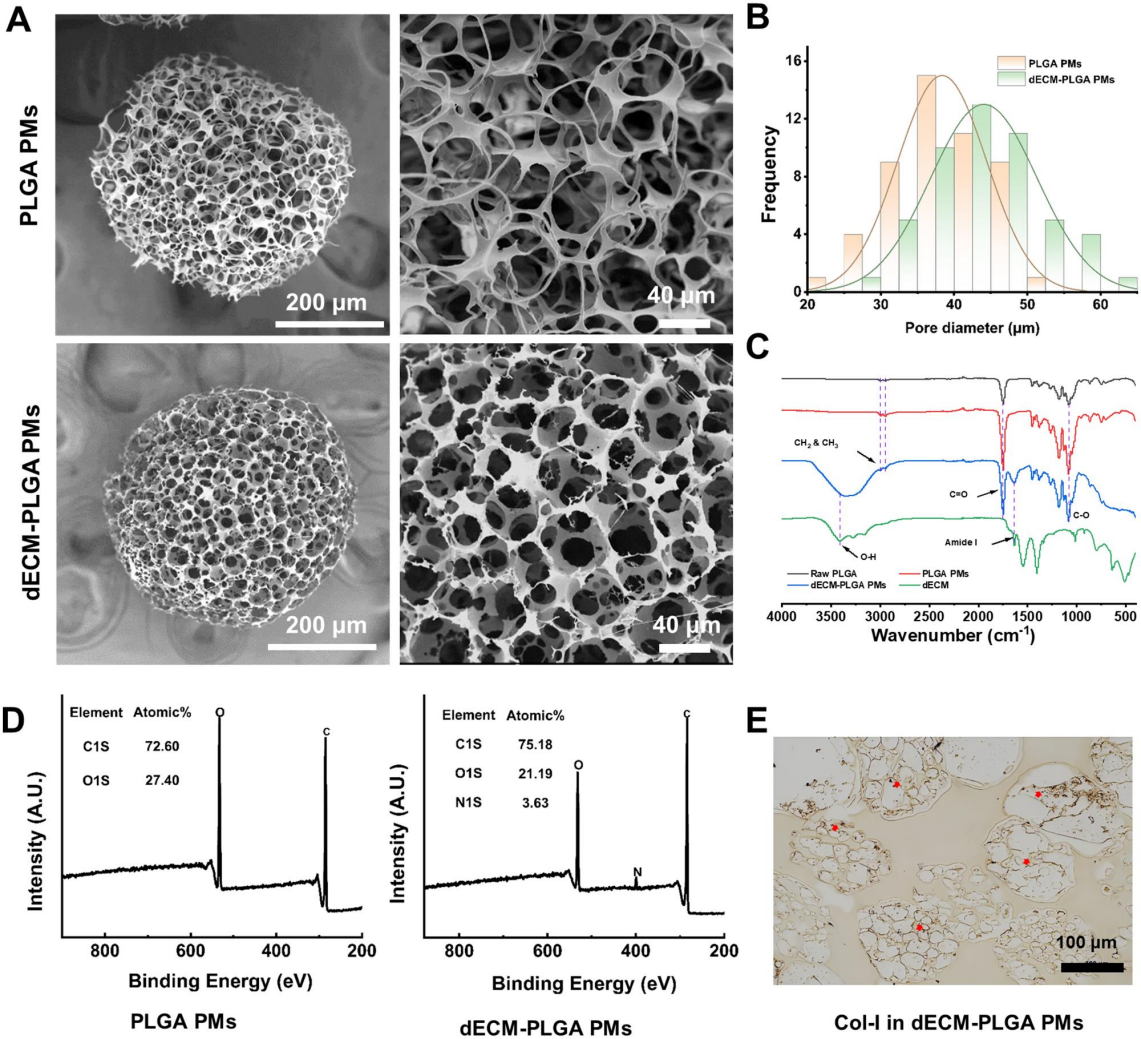
Characterization of dECM-PLGA PMs. (A) SEM images of PLGA PMs (up) and dECM-PLGA PMs (down). Scale bar = 200 μm (holistic view, left) and 40 μm (magnified view, right). (B) Pore size distribution of fabricated PMs based on SEM images. (C) FTIR analysis of various PMs based on attenuated total reflectance protocol. (D) XPS results of samples. XPS peaks of the PLGA PMs and dECM-PLGA PMs. (E) Immunohistochemical image of Collagen-I enriched in dECM-PLGA PMs showing the existence of liver-derived dECM. The arrow marked the positive components of dECM. Scale bar = 200 μm.

**Figure 4.**
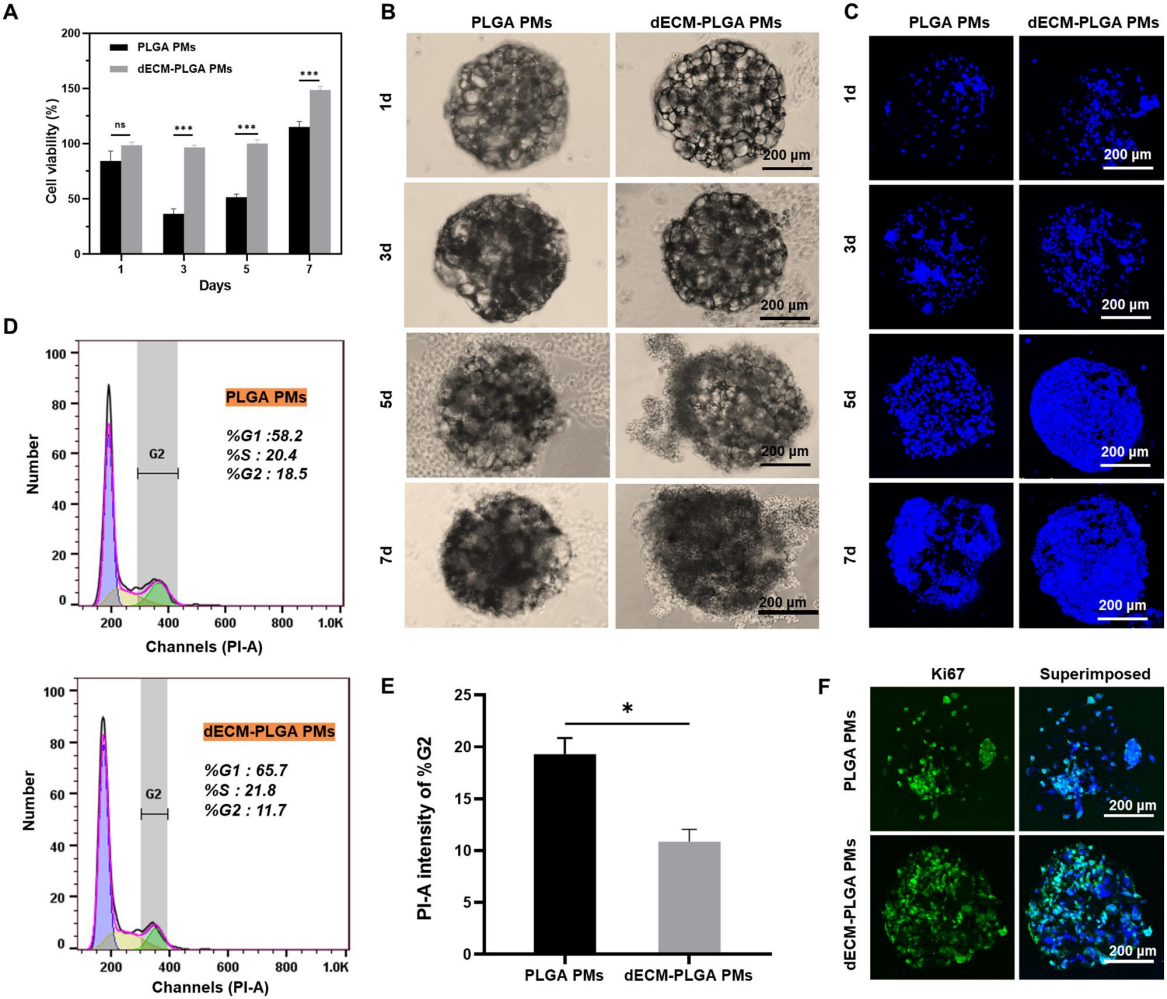
Proliferation of HepG2 on the dECM-PLGA PMs. (A) Cell viability of PMs-based microtumors for various cultural time. ***P < 0.001. Representative images of bright-field (B) and fluorescence images (C) showing the adhesion of HepG2 incubated PLGA PMs and dECM-PLGA PMs under dynamic culture for various cultural times. Scale bar = 200 μm. (D) Flow cytometry analysis of cell cycle variations of HepG2 adhesion with PLGA PMs and dECM-PLGA PMs (3 d). (E) The percentage of the G2 phase shows the proliferative activity of HepG2 adhesion on PLGA PMs and dECM-PLGA PMs (3 d). *P < 0.05. (F) Immunofluorescence morphology of Ki-67 expression of HepG2 adhesion on the PLGA PMs and dECM-PLGA PMs (3 d). Scale bar = 200 μm.

To closely replicate the TME of liver-derived ECM, porcine liver tissue was processed into dECM, which could be capable of mimicking the biomedical cues and structural intricacies surrounding *in vivo* tumors. Sequential treatment with positively charged SDS and nonionic Triton-X-100 detergents was chosen for cell removal, leveraging their well-established efficacy in decellularization [25]. Subsequently, the obtained tissue underwent enzymatic degradation to yield a dECM suspension, facilitating its use in an emulsion within a droplet microfluidic platform (Figure 1A). The SEM images of the native ECM depicted a densely packed and heterogenous assortment of cellular components intermingled with the ECM. This complex arrangement highlighted the diverse morphologies of cells within the native tissue architecture (Figure 1B, b-1). In contrast, the dECM exhibited an organized and uniform fibrous network. Post-decellularization, the resulting matrix displayed a distinct fibrous topology with enhanced definition, indicating the intrinsic fibrous nature of ECM character without cells (Figure 1B, b-2). The efficient removal of cells was confirmed through fluorescent imaging of DAPI-labeled cell nuclei in the tissue, further substantiated by quantification of DNA content in dECM samples (Figure 1B, b-3 and b-4). The quantification analysis of nuclear DNA content in freeze-dried tissue further verified the success of the decellularization process, with levels measuring less than 50 ng/mg (Figure 1C).

**Figure 5.**
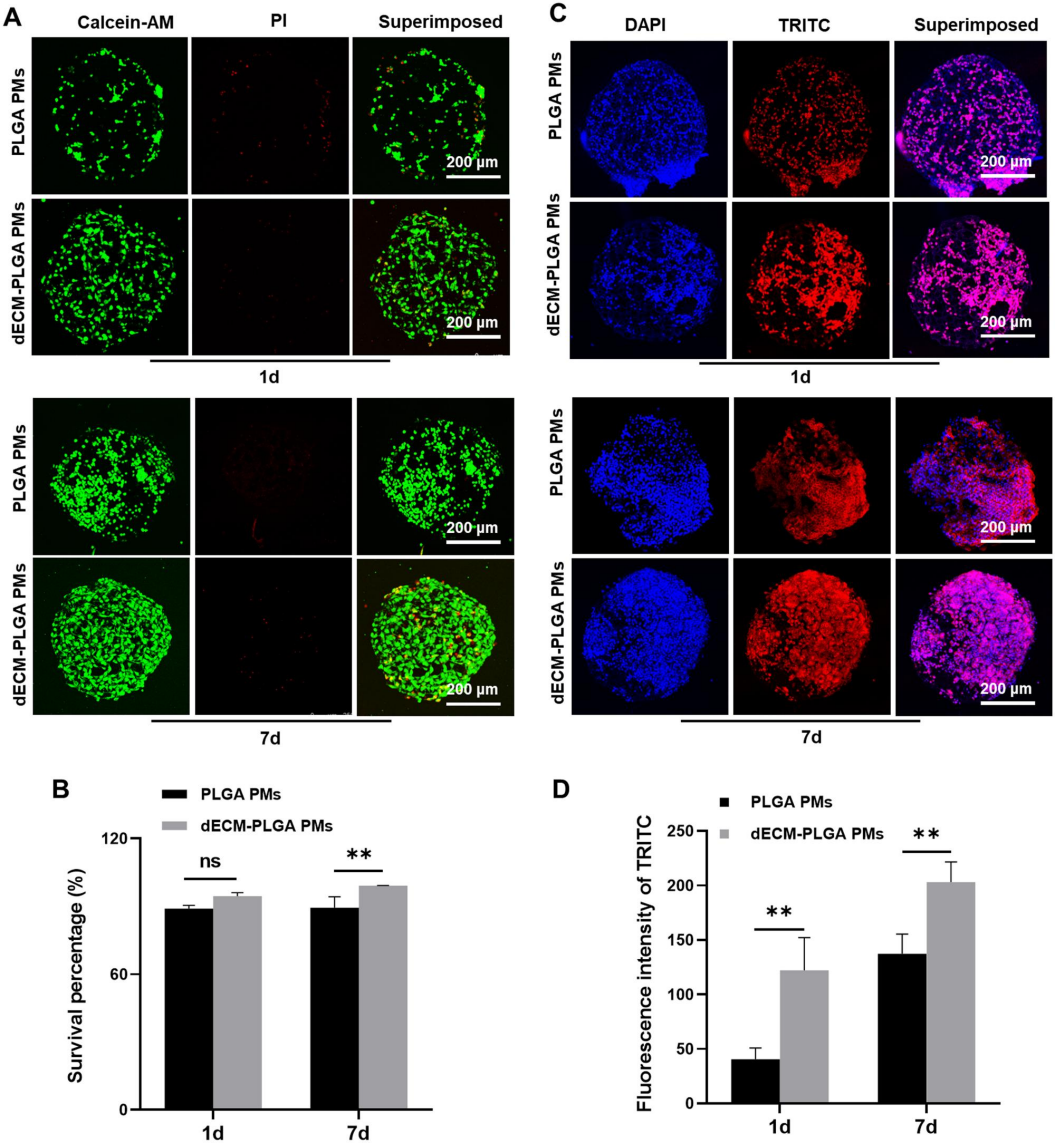
Evaluation of cell growth states on dECM-PLGA PMs. (A). CLSM images showing the cell viability of the PMs-based microtumors (1 d and 7 d). Scale bar =200 μm. (B) Survival percentage analysis of the ratio of live to dead stained HepG2. **P < 0.01. (C) CLSM images showing the cytoskeleton of HepG2 on the PMs (1 d and 7 d). Scale bar = 200 μm. (D) Fluorescence intensity analysis of TRITC positive stained HepG2. **P < 0.01.

**Figure 6.**
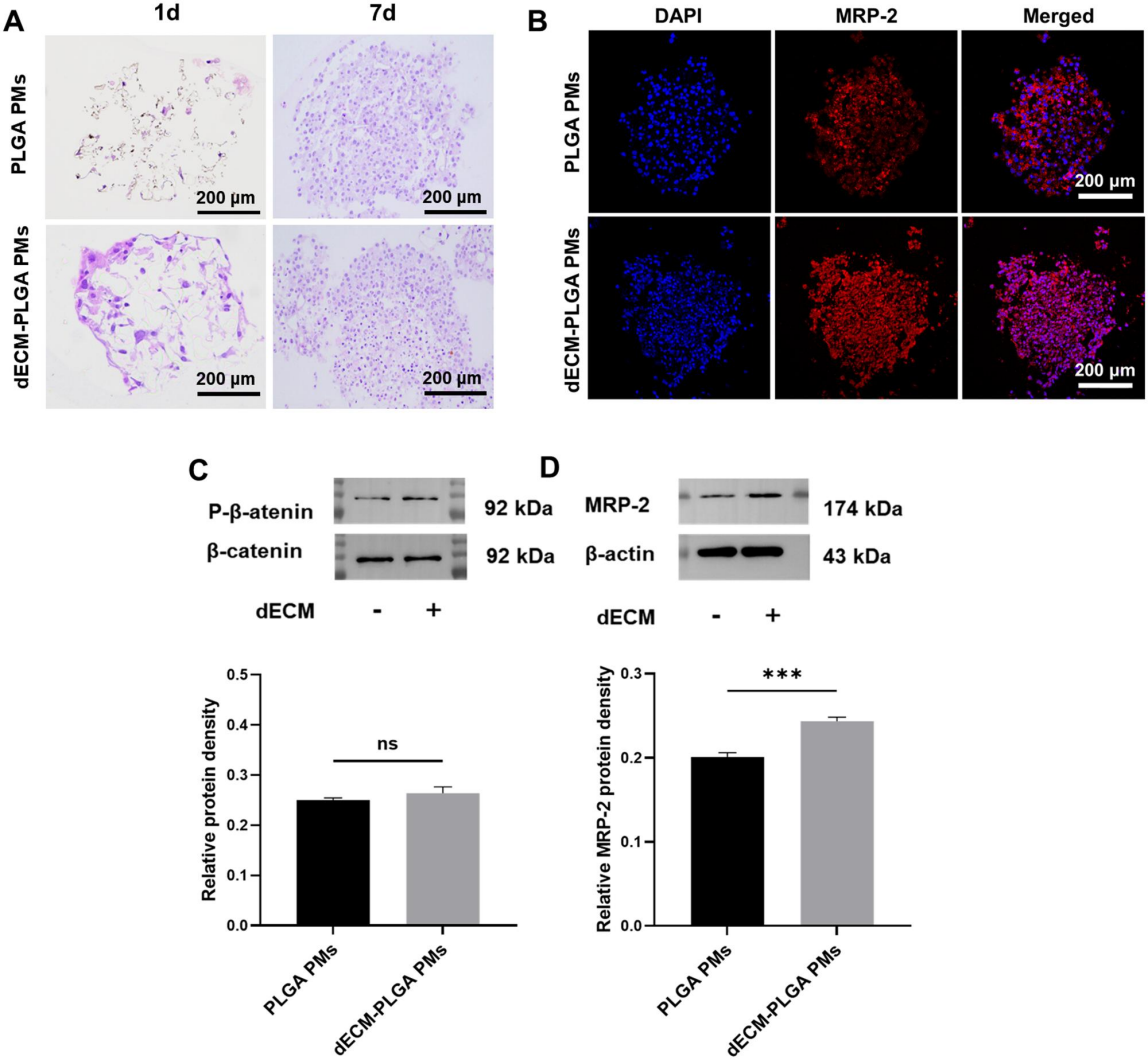
Evaluation of cell function on dECM-PLGA PMs. (A) H&E staining showing cell-laden PMs-based microtumors (1 d and 7 d). Scale bar = 200 μm. (B) Immunofluorescence morphology of MRP-2 expression of cell-laden PMs (7 d). Scale bar = 200 μm. (C) *p*-*β*-catenin/*β*-catenin, and (D) MRP-2 protein expression levels of PMs-based microtumors (10 d). ***P <* *0.001.

**Figure 7.**
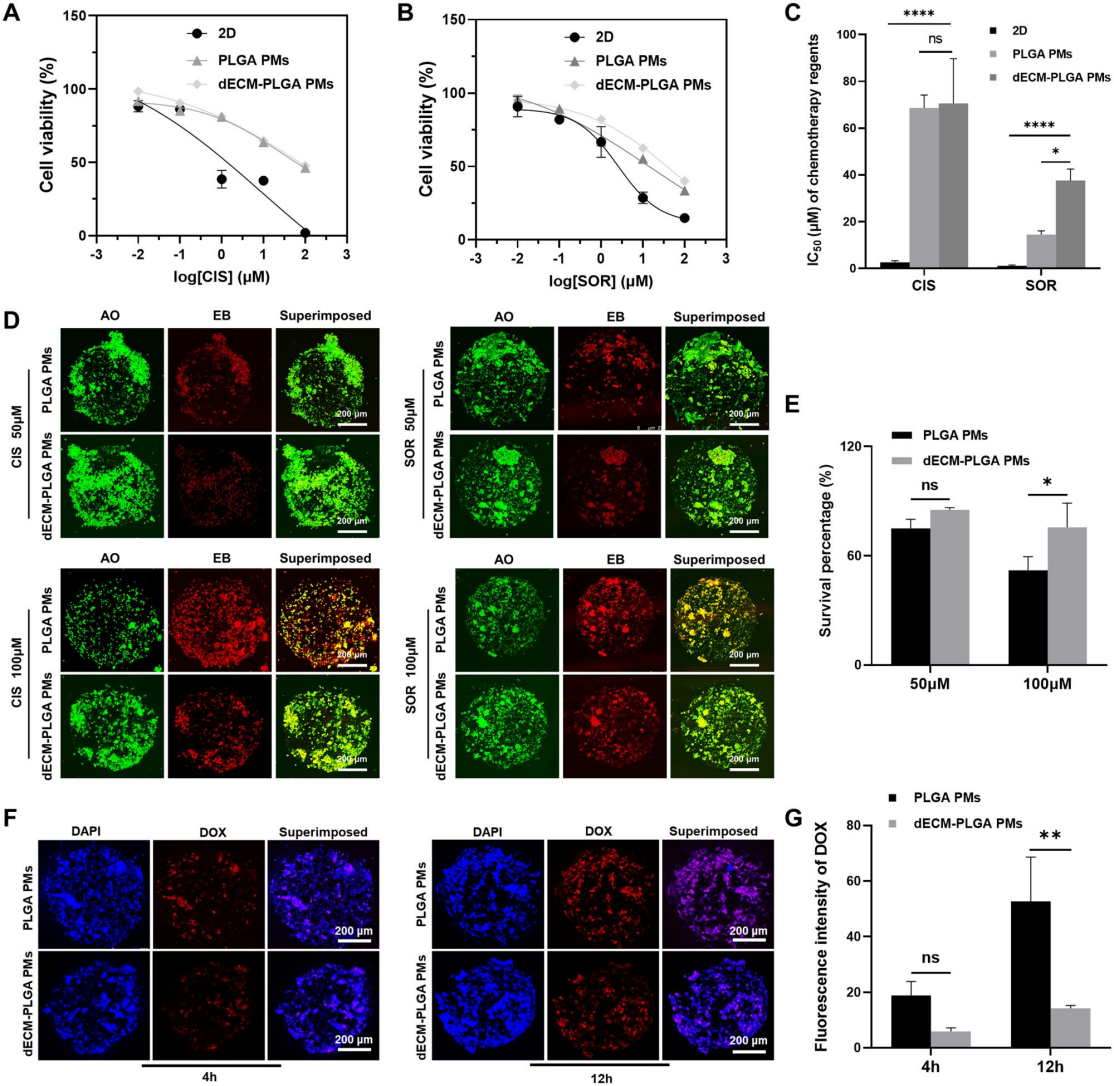
Drug evaluation on the dECM-PLGA PMs-based microtumors. (A, B) Drug susceptibility curve (CIS and SOR) *in vitro* showing the relative viabilities of HepG2 after incubation with different concentrations. (C) IC_50_ values analysis of anticancer drugs in different tumor models based on drug susceptibility curve. (D and E) Fluorescence microscopy analysis showing AO/EB dual staining of PMs-based microtumors after incubating with 50 and 100 μM CIS and SOR, respectively. Scale bar = 200 μm. (F and G) Fluorescence microscopy of cellular internalization of DOX partnered with DAPI staining in PMs-based microtumors.Scale bar = 200 μm.

H&E staining provided further evidence of the absence of cellular architecture in the dECM, as illustrated by the comparison. While the native tissue displayed a clear nucleo-cytoplasmic differentiation, this characteristic was notably absent in the dECM, which presented a more uniform histological appearance. This observation underscored the success of the decellularization protocol for the porcine liver, ensuring the removal of all cellular components while better preserving the structure of the ECM. Moreover, none of the visualizable cell debris or genetic components was retained (Figure 1B, b-5 and b-6). Masson’s trichrome staining indicates the preservation of collagen fibers in the dECM. In native liver tissue, collagen fibers coexist with cells, contributing to a complex tissue structure. However, in the dECM, although the collagen fibers were retained, the absence of cells results in a more open and discernible structure (Figure 1B, b-7 and b-8). Furthermore, Collagen-I and GAGs content in the liver dECM were quantified using enzyme-linked immunosorbent assay. The results indicated an increase in collagen content and a decrease in GAGs following decellularization (Figure 1D and E), highlighting the effectiveness of the employed removal methods.

### Proteomic analysis of porcine liver-derived dECM

The porcine liver-derived dECM underwent lyophilization and subsequent grinding into powder, followed by the treatment with pepsin to form a soluble powder for proteomic analysis. liquid chromatography–tandem mass spectrometry (LC–MS/MS) analysis, utilizing a label-free protein quantification approach, was used to estimate the absolute amount of protein amounts as established in the literature [26]. Kyoto Encyclopedia of Genes and Genomes (KEGG) and Gene Ontology (GO) analyses were conducted without a control sample to provide functional annotations for proteins based on similarity to known entities. This approach aided in understanding the biological processes, molecular functions and cellular localization by comparing them to information in databases and discovering associated biological pathways for gaining insights into the functional roles of proteins.

The porcine liver-derived dECM harbored diverse types of proteins, with the relative abundance of core ECM proteins ranked to provide an in-depth description of the dECM profiles in healthy porcine liver tissue (Supplementary Tables S1–S3). The results underscored the significant applied value of dECM architectures and composition in mimicking the liver’s natural environment and facilitating effective tumor model construction. Furthermore, the molecular weight distribution and sequence distribution length based on compositions in the liver-derived dECM were analysed, revealing average values of 66.53 kDa and 601.27, respectively (Figure 2A). These findings highlighted a controllable factor for many variations in this type of biomaterial. By classifying the identified proteins into these secondary classes, insights into major functional categories active in the sample can be gained. For example, many proteins in ‘Carbohydrate metabolism’ suggested active energy production processes (Figure 2B). Additionally, GO terms provided a high-level overview of the functions and locations of the proteins identified in the LC–MS/MS analysis, further enhancing understanding of their biological roles in biological processes, molecular function and cellular components (Figure 2C).

### Fabrication of dECM-PLGA PMs

To determine the optimal preparation parameters of composite PMs, three-factor and two-level full-factorial experiments were designed, which primarily investigated the effects of dECM concentration, gelatin concentration and ultrasonic power on the preparation outcomes. Herein, we kept the PLGA concentration fixed, ensuring a stable scaffold, and utilized pepsin-treated dECM mixed with a gelatin aqueous phase at a concentration optimized using a Minitab experimental design. As shown in Supplementary Table S4, the concentrations of dECM, gelatin and ultrasonic power were designated as factors influencing the structure of dECM-PLGA PMs, with pore size and particle size optimization as key parameters in the experimental design. The representative results were recorded after data collection in various groups in Supplementary Table S5, respectively. The resultant morphology of the composite microspheres was observed *via* SEM images. Different combinations of these factors influenced the microspheres obtained, with the output of software analysis consistently reflecting the overall morphology and structural changes of the microparticles (Supplementary Figure S1). These findings indicated a significant effect of ultrasonic power in conjunction with gelatin (low and high levels of the experimental design, *P* < 0.05), whereas no influence was observed on pore size. Additionally, the particle size of dECM-PLGA PMs was significantly affected by the concentration of dECM and gelatin (*P* < 0.05). The occupied space by porogen and dECM led to an increase in unit volume of the loading rate, resulting in an average pore size increase and a reduction in particle size (Supplementary Figure S2).

Furthermore, several factors, such as the components of droplets (disperse phase) at the tail end of the capillary tube, the sheer force of the continuous phase and water-in-oil (W/O) interfacial tension played a crucial role in this composite microsphere formation, as confirmed in previous results [21]. The objective of achieving dECM-PLGA PMs was characterized by a high percentage of dECM and highly open, interconnected cavities to facilitate cell adhesion and proliferation. The medium level, as stated in Supplementary Table S4, was selected as subsequent experimental formulation parameters (run 9) for co-culturing with HepG2. These optimized parameters included a PLGA concentration of 2% (w/v) and a flow rate ratio (continuous phase:disperse phase = 2:0.08) in the microfluidic platform.

### Characterization of dECM-PLGA PMs

SEM images revealed morphological and structural changes resulting from the incorporation of porcine liver-derived dECM into the PLGA PMs scaffold. Notably, the incorporation of dECM appeared to have influenced the porosity, with defined pores appearing. The microsphere structure displayed slightly rough-textured surface, potentially advantageous for cell attachment and proliferation (Figure 3A). Moreover, the pore size range of 20–60 μm could not only facilitate cell adhesion and inward migration, providing ample space for cells to spread and proliferate but also optimize the effective exchange of nutrients and waste, which was crucial for maintaining cellular metabolic activity and establishing a healthy cellular environment (Figure 3B) [12, 23, 27]. FTIR spectroscopy revealed distinctive spectral characteristics for PLGA. A pronounced absorption at roughly 1749 cm^−1^ could indicate the carbonyl stretch within the ester bonds, a key feature for PLGA identification in composite materials. The ester linkage also exhibited C–O stretch vibrations at 1100 cm^−1^, which could assist in evaluating the configuration of ester components and interaction in the PMs matrix. Importantly, PLGA hybridized with dECM resulted in additional peaks, such as those from hydroxyl (OH) groups at 3416 cm^−1^, which could be potentially linked to –OH functionalities of dECM. In addition, a notable Amide I peak at around 1643 cm^−1^ could represent the presence of collagen structures from the porcine liver-derived dECM [25]. Peaks between 2980 and 2950 cm^−1^, due to methylene (–CH_2_) vibrations, further delineated the C–H stretching within the material. These features collectively played a pivotal role in verifying the stability of PLGA polymer, evaluating its structural integrity, and subsequently understanding its interaction with porcine liver-derived dECM, especially relevant in the context of further biomedical application (Figure 3C). Moreover, XPS analysis of the surface chemical compositions of the PMs revealed elemental peaks of C 1s and O 1s for all PMs, with new nitrogen (N 1s) peaks detected in the spectra of dECM-PLGA PMs compared to PLGA PMs (Figure 3D). These results also confirmed that the porcine liver-derived dECM was successfully encapsulated in the PLGA skeleton. To illustrate the presence of bioactive components in dECM-PLGA PMs, the Col-I was stained using an immunochemical protocol. The immunohistochemical analysis demonstrated the identification of Collagen-I in sections of the composite PMs, with staining indicating a more uniform distribution across the porous structure of the microspheres (Figure 3E).

Prior to co-culturing with cells, we conducted investigations into the degradation of the PMs in PBS buffer to validate the release behavior of encapsulated dECM in the PLGA skeleton. Results depicted in Supplementary Figure S3 demonstrate a significant decrease in the pH of PBS over time, reaching levels comparable to PLGA PMs with increasing incubation time. Additionally, the surface of the PMs exhibited partial fragmentation as soluble ECM components were effectively liberated from the PLGA structure within one week (Supplementary Figure S4). Furthermore, scaffold degradation over several weeks *in vitro* was examined to compare the changes in the morphology. The dECM-PLGA PMs, characterized by a high loading rate (23.07% ± 1.45%), showed significant disintegration compared to PLGA PMs. Moreover, the compatibility of dECM-PLGA PMs was assessed through the CCK-8 assay and Calcein-AM and PI staining strategies. As depicted in Supplementary Figures S5 and S6, the viability of L929 cells at diverse leach liquor of dECM-PLGA PMs was maintained above 90% in all conditioned groups at the back of 48 h. The highest leach liquor at a concentration of 2 mg/ml was chosen to observe the status of L929 cells, as well as cellular morphology visualized by Calcein-AM staining. Compared to the control and PLGA PMs, the dECM-PLGA PMs presented a slight effect on L929 cells, including the cell morphology and survival state. Together, these findings indicated successful compounding of porcine liver-derived dECM into a PLGA skeleton, with the microarchitectures maintaining excellent viability for cells.

### Proliferative activity of HepG2 on the dECM-PLGA PMs

After biofunctionalization with porcine liver-derived dECM, the viability of the HepG2 both on the surface and within the fabricated cell-laden PMs was depicted (Figure 4A). The cell proliferation ability, assessed by CCK-8 assay, demonstrated minimal decrease over time for both PMs-based microtumors. Particularly, the PLGA PMs-based microtissues exhibited relatively lower cell viability compared to incubation with microtissues constructed with dECM-PLGA PMs. The discrepancy suggested that an ECM-relative component could significantly influence cell viability, as indicated by statistical analysis.

Further, the adhesion of HepG2 cells was evaluated, and the cell number on individual PMs was counted at various time points (Figure 4B). A noticeable increase in cell number over time was observed for both scaffolds, with dECM-PLGA PMs exhibiting a substantially higher cell adhesion rate over other group. Moreover, HepG2 tended to adhere to the surface of dECM-PLGA PMs, with many cells maintaining an even distribution on the PMs, facilitating tight connections (Figure 4C). The observed deviation in cell distribution between the two scaffolds corresponds well with the differences in cell viability, indicating that biofunctionalized PMs could provide a suitable environment akin to the ECM *in vivo*, promoting adhesion and viability.

FCM and IF analyses were conducted to evaluate the properties of proliferation activity in depth (Figure 4D and E). The cell cycle analysis revealed a higher abundance of cells in the G1 and S phases relative to the G2 phase, indicating that cells were dedicating more resources to DNA synthesis rather than mitosis. Notably, the G2 proportion of dECM-PLGA PMs-based microtissues was lower compared to PLGA PM-based microtissues, suggesting that the cells were in a state of rising proliferative activity. To corroborate these findings, Ki-67, a protein associated with various stages of the cell cycle, particularly late G1, S, G2 and M-phase, was visualized using CLSM (Figure 4F). The protein encoded a nuclear protein, which was expressed amid diverse stages in the cell cycle, particularly during the late G1, S, G2 and M-phase [28]. The representative images revealed an abundance of positive signals in the cell-laden dECM-PLGA PMs, indicating enhanced effectiveness of cell proliferation facilitated by the bioactive components.

### Construction and assessment of *in vitro* 3D hepatic model

#### Evaluation of cell growth states on dECM-PLGA PMs

To explore the functionality of these *in vitro* 3D hepatic models, both PLGA PMs and dECM-PLGA PMs were employed for co-culturing with HepG2 for 7 days to form cohesive cellular microtissues. Live and dead cell analysis of the cell-laden PMs was measured using CLSM to evaluate further the function of the cell-laden PMs. The obtained data revealed that both PLGA PMs and dECM-PLGA PMs supported cell proliferation. However, distinct results were observed in the cell-laden dECM-PLGA PMs; tumor microtissues appeared denser by day 7, while cellular distribution within PLGA PMs showed uneven growth (Figure 5A). Further by semi-quantitative analysis of the fluorescence results, a high percentage of cell survival was maintained on both the first and seventh day of co-culture with HepG2, indicating the better biocompatibility of the two porous microspheres. The dECM component in the composite porous microsphere structure exerted a significant cell growth-promoting effect as the incubation time increased (Figure 5C, *P* < 0.01). The discrepancy suggested that the presence of dECM could facilitate the formation of cell aggregation.

Furthermore, the cytoskeletal organization was visualized using TRITC-phalloidin staining in both PLGA PMs and dECM-PLGA PMs. Notably, the tight morphological properties of cells were more evident in dECM-PLGA PMs. The integration of tight cellular interactions played a crucial role in simulating the natural *in vivo* hepatic tissue environment for HepG2, thereby enhancing the accuracy in evaluating drug effects. Including tight junctions within the microtissues significantly strengthened the barrier function, which was vital for maintaining specific biochemical environments that affected drug effectiveness and metabolism in liver cancer. Additionally, the tight connections between cells ensured that structural and functional integrity was preserved, which boosted the reliability and reproducibility of our drug response assessments (Figure 5C). The porcine liver-derived dECM component was able to promote the adhesion of hepatocellular carcinoma cells on the surface of the porous microsphere at the initial stage of co-culture of microspheres with HepG2. With the prolongation of the co-culture time and the continued role of the dECM components in the microsphere structure, this effect of promoting cell adhesion and the formation of tight cell junctions remained significant (Figure 5D). Thus, the dECM-PLGA PMs-based tumor model could effectively replicate the TME found in liver cancer, providing a solid platform for the accurate and applicable evaluation of potential cancer therapies and their safety profiles.

#### Evaluation of cell function on dECM-PLGA PMs

To evaluate the HepG2 on the PMs, the histological examination *via* H&E staining revealed that HepG2 exhibited a marked affinity for liver-derived dECM, evidently from the significant cell adhesion on dECM-PLGA PMs as early as day 1 in a comparative analysis (Figure 6A). HepG2 cells exhibited enhanced adhesion and proliferation on dECM-PLGA PMs compared to PLGA PMs, which was evidenced by a more uniform distribution and increased cell density after 7 days. These findings suggested that the addition of dECM facilitated a biomimetic environment that promoted better cell–matrix interactions.

Furthermore, MRP-2, a member of the ATP-binding cassette transporter family, plays a crucial role in expelling chemotherapeutic agents from cancer cells. This drug efflux pump contributes to multidrug resistance, significantly reducing the efficacy of chemotherapy. The overexpression of MRP-2 is commonly associated with poor chemotherapy outcomes across various cancers [29]. HepG2 cultured on PLGA and dECM-PLGA microsphere scaffolds underwent staining to evaluate the expression of MRP-2, identified by red fluorescence (Figure 6B). Cells on PLGA scaffolds showed localized MRP-2 expression, which could indicate confined capabilities for drug efflux in environments without dECM. In contrast, cells on dECM-PLGA PMs revealed a more extensive and intense MRP-2 expression pattern. The enhanced expression of MRP-2 in the presence of dECM might reflect an environment that could closely mimic physiological conditions. The expression pattern was indicative of the crucial role dECM played in supporting liver-specific functions, with elevated MRP-2 levels possibly signifying increased metabolic activity or heightened responsiveness to chemotherapeutic compounds, which was of considerable importance for understanding the drug response mechanisms in liver cancer cells.

Moreover, the related protein expression level was also used to understand the difference between dECM-PLGA PMs and PLGA PMs at 3D conditions. A slight rise in the levels of the ratio of *β*-catenin to phosphorylated *β*-catenin was observed, indicating that the presence of dECM might not influence the activation state of the Wnt signaling pathway (Figure 6C) [30]. However, notable upregulation of the MRP-2 protein was observed in the presence of dECM, which suggested that dECM provided a supportive microenvironment that enhanced cellular mechanisms involved in drug resistance and efflux, key functions of liver cells in detoxification (Figure 6D). Together, the microenvironment created by the dECM-PLGA PMs might enhance transport protein expression, underscoring the complexity of this liver-specific ECM effected on cellular behavior in 3D cultures.

### Drug sensitivity testing

During preclinical screening of anticancer drugs, cytotoxicity assays provided valuable insights into the drug effects on various tumor cells, and evaluating the efficacy of anticancer drugs in various cell culture models is crucial for understanding their potential clinical impact [6, 31]. We investigated the cytotoxic effects of CIS and SOR on HepG2 under different conditions: conventional 2D tumor model, 3D tumor model based on PLGA PMs and dECM-PLGA PMs. The dose–response curves for CIS showed a decline in cell viability across all models with increasing drug concentrations. However, cells cultured in dECM-PLGA PMs exhibited comparatively higher viability at higher concentrations, suggesting enhanced drug resistance (Figure 7A). Similar trends were observed with SOR, where all models displayed decreased cell viability with increasing concentrations of the drug. Notably, the dECM-PLGA PMs-based 3D model demonstrated the highest cell viability, particularly at higher drug concentrations, indicating significant resistance to SOR compared to the 2D and PLGA PMs model (Figure 7B). The calculated IC_50_ values further substantiate the observations, with dECM-PLGA PMs showing the highest IC_50_ values for both drugs, indicating the lowest sensitivity (Figure 7C). These results underscored the significant impact of the cellular microenvironment on drug sensitivity, highlighting the importance of selecting an appropriate model for drug screening. dECM-PLGA PMs offered a closer approximation to the native liver environment, potentially providing more accurate predictions of drug responses *in vivo*. Such 3D culture systems could be crucial for developing more effective therapeutic strategies, reflecting the true clinical potential of anticancer drugs. The integration of dECM in 3D PLGA microspheres enhanced cell–matrix interactions, thus impacting drug distribution within the microenvironment and contributing to the drug resistance previously observed in the tumor cells, such as Phosphoinositide 3-kinases/Protein Kinase B (PI3K/Akt) and Mitogen-Activated Protein Kinases (MAPK) [20, 32]. These results emphasized the importance of using dECM-enhanced 3D PM-based models to simulate *in vivo* tumor behavior more accurately than PMs in drug screening.

Furthermore, the cytotoxic sensitivity of HepG2 to CIS and SOR was analysed using AO/EB dual staining under 3D culture conditions facilitated by PLGA PMs and dECM-PLGA PMs at concentrations of 50 and 100 μM. The AO/EB staining distinguished viable cells emitting green fluorescence (AO) from dead cells showing red fluorescence (EB), with early apoptotic cells appearing yellow due to the superposition of the stains. For both CIS and SOR treatments, HepG2 cultured in dECM-PLGA PMs consistently displayed higher green fluorescence intensity across both concentrations compared to those in PLGA PMs, indicating a higher proportion of viable cells. The differential survival observed was more pronounced at the higher concentration of 100 μM, where dECM-PLGA PMs exhibited fewer red fluorescence, suggesting lower cell death and apoptosis rate (Figure 7D). Moreover, the survival percentages were calculated to analyse the drug sensitivity. The difference between the two PMs was not statistically significant at 50 μM, but the survival rates in dECM-PLGA PMs were notably higher than those in PLGA PMs, with statistical significance at 100 μM. This substantiates the enhanced resistance to cytotoxic effects in dECM-PLGA PMs, particularly under higher drug concentrations (Figure 7E). The fluorescence images and the semi-quantitative fluorescence data both underscored the reliability of enhanced cell survival in the dECM-PLGA PMs,corroborating the hypothesis that the dECM within the PMs created a more favorable microenvironment and potentially mimicking the natural ECM and thereby enhancing cellular resistance to chemotherapy agents.

Meanwhile, DOX is an ideal model drug for cancer research due to its intrinsic fluorescent properties, which facilitate direct observation and quantification of drug uptake and distribution within cells. The natural fluorescence of DOX eliminates the need for additional labeling, simplifies experimental procedures and increases the reliability of data. This feature is essential in investigating drug interactions and behaviors within complex biological systems, such as liver cancer cells embedded in various scaffold compositions [33, 34]. Considering this attribute, the red fluorescence of DOX was utilized to assess drug uptake in HepG2 cultured within PLGA PMs and dECM-PLGA PMs. Both PLGA PMs and dECM-PLGA PMs showed enhanced red fluorescence, in which the intensity and distribution were pronounced with the incubation time. Compared to the fluorescence intensity in PLGA PMs, dECM-PLGA PMs showed lower DOX internalization (Figure 7F). Similar trends were further semi-quantitatively analysed with Image J software, of which PLGA PMs were slightly higher than in dECM-PLGA PMs at 4 h, and the fluorescence intensity of dECM-PLGA PMs-based microtumors significantly lower than that in PLGA PMs by 12 h (Figure 7G). Together, the significant differences in drug uptake and retention at different time points underscored the importance of the microenvironment in influencing drug response in 3D tumor models.

## Conclusion

In summary, dECM-based 3D tumor models offered a promising approach to constructing a more biomimetic TME model *in vitro*. Considering that the existing models lacked the assembled morphology necessary for tumor assembly during cell adhesion stages, we established a protocol that incorporated with porcine liver-derived dECM fragments into PLGA PMs, thereby enhancing the *in vivo*-like environment and reproducing key organotypic cellular and matrix components of the TME. By co-culturing HepG2 with dECM-PLGA PMs and employing a bottom-up approach, 3D microtumors under dynamic culture conditions were morphologically defined. The dECM-PLGA PMs-based microtumors, characterized by random cell distribution, represented a significant advancement in tumor drug screening. In terms of applicability, two chemotherapeutic drugs, namely CIS and SOR, revealed that dECM-PLGA PMs-based microtumors exhibited higher-level drug resistance. These findings could be attributed to the thorough enrichment of key organotypic hallmarks facilitated by the incorporation of dECM. Therefore, scaffold-based microtumors presented an attractive platform for studying drug mechanisms, identifying new drug targets, and expediting the screening of anticancer candidate drugs. However, it is challenging to replicate the intricate heterogeneity of *in vivo* tumors comprehensively. To address this aspect, future work will be focused on refining the model’s fidelity and integrating advanced positive controls to enhance its clinical relevance.

## Supplementary Material

rbae113_Supplementary_Data

## References

[1] Wakefield L , Agarwal S, Tanner K. Preclinical models for drug discovery for metastatic disease. Cell 2023;186:1792–813. 37059072 10.1016/j.cell.2023.02.026

[2] Thoma CR , Zimmermann M, Agarkova I, Kelm JM, Krek W. 3D cell culture systems modeling tumor growth determinants in cancer target discovery. Adv Drug Deliv Rev 2014;69–70:29–41. 10.1016/j.addr.2014.03.001 24636868

[3] Brancato V , Oliveira JM, Correlo VM, Reis RL, Kundu SC. Could 3D models of cancer enhance drug screening? Biomaterials 2020;232:119744. 31918229 10.1016/j.biomaterials.2019.119744

[4] Ma JN , Dai LS, Yu JB, Cao H, Bao YM, Hu JJ, Zhou LH, Yang JQ, Sofia A, Chen HW, Wu F, Xie ZK, Qian WQ, Zhan RY. Tumor microenvironment targeting system for glioma treatment via fusion cell membrane coating nanotechnology. Biomaterials 2023;295:122026. 36731366 10.1016/j.biomaterials.2023.122026

[5] Zhang CY , Fu CP, Li XY, Lu XC, Hu LG, Kankala RK, Wang SB, Chen AZ. Three-dimensional bioprinting of decellularized extracellular matrix-based bioinks for tissue engineering. Molecules 2022;27:3442. 35684380 10.3390/molecules27113442 PMC9182049

[6] Ferreira LP , Gaspar VM, Mendes L, Duarte IF, Mano JF. Organotypic 3D decellularized matrix tumor spheroids for high-throughput drug screening. Biomaterials 2021;275:120983. 34186236 10.1016/j.biomaterials.2021.120983

[7] Tao WY , Tuo Z, Wu FG, Mu KT, Xu CJ, Shi YX, Sun ZY, Wang YF, Li Y, Zhong ZY, Zhou L, Wang JL, Liu J, Du YY, Zhang SM. Albumin-assembled copper-bismuth bimetallic sulfide bioactive nanosphere as an amplifier of oxidative stress for enhanced radio-chemodynamic combination therapy. Regen Biomater 2022;9:rbac045. 35855112 10.1093/rb/rbac045 PMC9290530

[8] Junttila MR , de Sauvage FJ. Influence of tumour microenvironment heterogeneity on therapeutic response. Nature 2013;501:346–54. 24048067 10.1038/nature12626

[9] Wang SY , Yu HJ, Wan GS, Fang HW, Mi JX, Xu WQ, Sun KX, Zhang KX, Yin JB, Deng WL. Highly tough and elastic microspheric gel for transarterial catheter embolization in treatment of liver metastasis tumor. Regen Biomater 2023;10:rbad026. 37016664 10.1093/rb/rbad026 PMC10067152

[10] Liu YL , Yang XQ, Jiang D, Hu RC, Huang FL, Zou XN, Liu C, Peng ZW. 3D biomimetic tumor microenvironment of HCC to visualize the intercellular crosstalk between hepatocytes, hepatic stellate cells, and cancer cells. Smart Mater Med 2023;4:384–95.

[11] Zhu LY , Yuhan J, Yu H, Zhang BY, Huang KL, Zhu LJ. Decellularized extracellular matrix for remodeling bioengineering organoid's microenvironment. Small 2023;19:e2207752. 36929582 10.1002/smll.202207752

[12] Wang Y , Kankala RK, Zhang JT, Hao LZ, Zhou K, Wang SB, Zhang YS, Chen AZ. Modeling endothelialized hepatic tumor microtissues for drug screening. Adv Sci 2020;7:2002002. 10.1002/advs.202002002 PMC7610277 33173735

[13] Shie MY , Fang HY, Kan KW, Ho CC, Tu CY, Lee PC, Hsueh PR, Chen C-H, Lee AKX, Tien N, Chen JX, Shen YC, Chang JG, Shen YF, Lin TJ, Wang B, Hung MC, Cho DY, Chen YW. Highly mimetic ex vivo lung-cancer spheroid-based physiological model for clinical precision therapeutics. Adv Sci 2023;10:e2206603. 10.1002/advs.202206603 PMC10238206 37085943

[14] Xu PY , Kankala RK, Wang SB, Chen AZ. Decellularized extracellular matrix-based composite scaffolds for tissue engineering and regenerative medicine. Regen Biomater 2024;11:rbad107. 38173774 10.1093/rb/rbad107 PMC10761212

[15] Xu PY , Cao JT, Duan YY, Kankala RK, Chen AZ. Recent advances in fabrication of dECM-based composite materials for skin tissue engineering. Front Bioeng Biotechnol 2024;12:1348856. 38322790 10.3389/fbioe.2024.1348856 PMC10844517

[16] Zhang J , Si RJ, Gao Y, Shan H, Su Q, Feng ZL, Huang PS, Kong DL, Wang WW. dECM restores macrophage immune homeostasis and alleviates iron overload to promote DTPI healing. Regen Biomater 2024;11:rbad118. 38404617 10.1093/rb/rbad118 PMC10884736

[17] Kim W , Lee H, Lee J, Atala A, Yoo JJ, Lee SJ, Kim GH. Efficient myotube formation in 3D bioprinted tissue construct by biochemical and topographical cues. Biomaterials 2020;230:119632. 31761486 10.1016/j.biomaterials.2019.119632 PMC7141931

[18] Jung M , Han Y, Woo C, Ki CS. Pulmonary tissue-mimetic hydrogel niches for small cell lung cancer cell culture. J Mater Chem B 2021;9:1858–66. 33533364 10.1039/d0tb02609c

[19] Lv WK , Zhou HZ, Aazmi A, Yu MF, Xu XB, Yang HY, Huang YYS, Ma L. Constructing biomimetic liver models through biomaterials and vasculature engineering. Regen Biomater 2022;9:rbac079. 36338176 10.1093/rb/rbac079 PMC9629974

[20] Weigelt B , Ghajar CM, Bissell MJ. The need for complex 3D culture models to unravel novel pathways and identify accurate biomarkers in breast cancer. Adv Drug Deliv Rev 2014;69–70:42–51. 10.1016/j.addr.2014.01.001 PMC4186247 24412474

[21] Kankala RK , Zhao J, Liu CG, Song XJ, Yang DY, Zhu K, Wang SB, Zhang YS, Chen AZ. Highly porous microcarriers for minimally invasive in situ skeletal muscle cell delivery. Small 2019;15:e1901397. 31066236 10.1002/smll.201901397 PMC6750270

[22] Luo SC , Wang Y, Kankala RK, Zhang YS, Chen AZ. Fabricating highly open porous microspheres (HOPMs) via microfluidic technology. J Vis Exp 2022;183:63971. 10.3791/63971 35635463

[23] Wang Y , Kankala RK, Cai YY, Tang HX, Zhu K, Zhang JT, Yang DY, Wang SB, Zhang YS, Chen AZ. Minimally invasive co-injection of modular micro-muscular and micro-vascular tissues improves in situ skeletal muscle regeneration. Biomaterials 2021;277:121072. 34454373 10.1016/j.biomaterials.2021.121072

[24] Lashen AG , Toss MS, Ghannam SF, Makhlouf S, Green A, Mongan NP, Rakha E. Expression, assessment and significance of Ki67 expression in breast cancer: an update. J Clin Pathol 2023;76:357–64. 36813558 10.1136/jcp-2022-208731

[25] Ergun C , Parmaksiz M, Vurat MT, Elcin AE, Elcin YM. Decellularized liver ECM-based 3D scaffolds: compositional, physical, chemical, rheological, thermal, mechanical, and in vitro biological evaluations. Int J Biol Macromol 2022;200:110–23. 34971643 10.1016/j.ijbiomac.2021.12.086

[26] Qiu PC , Li MB, Chen K, Fang B, Chen PF, Tang ZB, Lin XF, Fan SW. Periosteal matrix-derived hydrogel promotes bone repair through an early immune regulation coupled with enhanced angio- and osteogenesis. Biomaterials 2020;227:119552. 31670079 10.1016/j.biomaterials.2019.119552

[27] Chen Y , Chen LF, Wang Y, Duan YY, Luo SC, Zhang JT, Kankala RK, Wang SB, Chen AZ. Modeling dECM-based inflammatory cartilage microtissues in vitro for drug screening. Compos. Part B Eng 2023;250:110437.

[28] Sensi F , D'Angelo E, Biccari A, Marangio A, Battisti G, Crotti S, Fassan M, Laterza C, Giomo M, Elvassore N, Spolverato G, Pucciarelli S, Agostini M. Establishment of a human 3D pancreatic adenocarcinoma model based on a patient-derived extracellular matrix scaffold. Transl Res 2023;253:57–67. 36096350 10.1016/j.trsl.2022.08.015

[29] Szakács G , Paterson JK, Ludwig JA, Booth-Genthe C, Gottesman MM. Targeting multidrug resistance in cancer. Nat Rev Drug Discov 2006;5:219–34. 16518375 10.1038/nrd1984

[30] Hong G , Kim J, Oh H, Yun S, Kim CM, Jeong YM, Yun WS, Shim JH, Jang I, Kim CY, Jin S. Production of multiple cell-laden microtissue spheroids with a biomimetic hepatic-lobule-like structure. Adv Mater 2021;33:e2102624. 34286875 10.1002/adma.202102624 PMC11469225

[31] Sun L , Wang XF, He YS, Chen BR, Shan BY, Yang JL, Wang RR, Zeng XH, Li JH, Tan H, Liang RC. Polyurethane scaffold-based 3D lung cancer model recapitulates in vivo tumor biological behavior for nanoparticulate drug screening. Regen Biomater 2023;10:rbad091. 37965109 10.1093/rb/rbad091 PMC10641150

[32] Ahn S , Sharma U, Kasuba KC, Strohmeyer N, Müller DJ. Engineered biomimetic fibrillar fibronectin matrices regulate cell adhesion initiation, migration, and proliferation via α5β1 integrin and syndecan-4 crosstalk. Adv Sci 2023;10:2300812. 10.1002/advs.202300812 PMC10460904 37357136

[33] Mazza T , Roumeliotis TI, Garitta E, Drew D, Rashid ST, Indiveri C, Choudhary JS, Linton KJ, Beis K. Structural basis for the modulation of MRP2 activity by phosphorylation and drugs. Nat Commun 2024;15:1983. 38438394 10.1038/s41467-024-46392-8 PMC10912322

[34] Minotti G , Menna P, Salvatorelli E, Cairo G, Gianni L. Anthracyclines: molecular advances and pharmacologic developments in antitumor activity and cardiotoxicity. Pharmacol Rev 2004;56:185–229. 15169927 10.1124/pr.56.2.6

