# Supplementary material for: Engineered liver-derived decellularized extracellular matrix-based three-dimensional tumor constructs for enhanced drug screening efficiency
Source: Regen Biomater. 2024 Sep 6;11:rbae113. doi: 10.1093/rb/rbae113 (PMC11441757; doi:10.1093/rb/rbae113)
Supplement: rbae113_Supplementary_Data [file rbae113_supplementary_data.zip › Supporting information.docx]

*Supporting information*

**Engineered Liver-derived Decellularized Extracellular Matrix-based Three-dimensional Tumor Constructs for Enhanced Drug Screening Efficiency**

*Shengchang Luo^a,b^, Qingqing Wang^a,b^, Miaoting Li^a,b^, Peiyao Xu^a,b^, Yicheng Wang^a,b^, Ying Wang* ^c d^, Ranjith Kumar Kankala^a,b^, ShiBin Wang^a,b^, Aizheng Chen*^,a,b^*

*^a^ Institute of Biomaterials and Tissue Engineering, Huaqiao University, Xiamen, 361021, P. R. China*

*^b^ Fujian Provincial Key Laboratory of Biochemical Technology (Huaqiao University), Xiamen, 361021, P. R. China*

*^c^ Institute of Biomedical Engineering, University of Toronto, Toronto, Ontario, M5S 3G9, Canada*

*^d^ Toronto General Research Institute, Toronto; Ontario, M5G 2C4, Canada*

**Corresponding authors*

*azchen@hqu.edu.cn (A. Z. Chen) Tel/fax: +86 592 616 2326*

*wangying277@outlook.com (Y. Wang)*

**Table S1.** Composition of collagens in porcine liver-derived dECM

| Rank | Gene name |  | LFQ (log10) | Protein name |
| --- | --- | --- | --- | --- |
| 1 | COL1A1 |  | 9.65 | Collagen type I alpha 1 chain |
| 2 | COL1A2 |  | 9.53 | Collagen type I alpha 2 chain |
| 3 | COL6A1 |  | 8.57 | Collagen type VI alpha 1 chain |
| 4 | COL4A |  | 8.56 | Collagen IV NC1 domain-containing protein |
| 5 | COL6A3 |  | 8.53 | Collagen type VI alpha 3 chain |
| 6 | COL4A2 |  | 8.40 | Collagen type IV alpha 2 chain |
| 7 | COL6A2 |  | 8.39 | Collagen type VI alpha 2 chain |
| 8 | COL6A5 |  | 8.21 | Collagen type VI alpha 5 chain |
| 9 | COL5A2 |  | 8.12 | Collagen type V alpha 2 chain |
| 10 | COL3A1 |  | 7.99 | Collagen alpha-1(III) chain preproprotein |
| 11 | COL5A1 |  | 7.97 | Collagen type V alpha 1 chain |
| 12 | COL18A1 |  | 7.49 | Collagen type XVIII alpha 1 chain |
| 13 | COL14A1 |  | 7.31 | Collagen type XIV alpha 1 chain |
| 14 | COL2A1 |  | 7.31 | Collagen type II alpha 1 chain |
| 15 | COL12A1 |  | 6.88 | Collagen type XII alpha 1 chain |
| 16 | COL6A3 |  | 6.76 | Collagen type VI alpha 3 chain |
| 17 | COL6A5 |  | 6.69 | Collagen type VI alpha 5 chain |
| 18 | COL6A6 |  | 6.60 | Collagen type VI alpha 6 chain |
| 19 | COL4A6 |  | 6.12 | Collagen type IV alpha 6 chain |

**Table S2.** Composition of growth factors and receptors in porcine liver-derived dECM

| Rank | Gene name | LFQ (log10) | Protein name |
| --- | --- | --- | --- |
| 1 | TNFRSF13B | 8.00 | TNF receptor superfamily member 13B |
| 2 | SCARF2 | 7.82 | Scavenger receptor class F member 2 |
| 3 | DDX58 | 7.40 | Antiviral innate immune response receptor RIG-I |
| 4 | RACK1 | 7.27 | Receptor of activated protein C kinase 1 |
| 5 | PGRMC1 | 7.03 | Membrane-associated progesterone receptor component 1 |
| 6 | LTBP1 | 6.95 | Latent transforming growth factor beta binding protein 1 |
| 7 | TGFBI | 6.91 | Transforming growth factor-beta-induced protein ig-h3 |
| 8 | TGFBI | 6.91 | Transforming growth factor-beta-induced protein ig-h3 |
| 9 | SRPRB | 6.89 | Signal recognition particle receptor subunit beta |
| 10 | SRPRA | 6.87 | SRP receptor subunit alpha |
| 11 | HDGF | 6.20 | Heparin-binding growth factor |
| 12 | EGFR | 5.84 | Receptor protein-tyrosine kinase |
| 13 | PEX5 | 5.76 | Peroxisomal targeting signal 1 receptor |
| 14 | RXRA | 5.51 | Retinoic acid receptor RXR |

**Table S3.** Composition of ECM glycoproteins in porcine liver-derived dECM

| Rank | Gene name | LFQ (log10) | Protein name |
| --- | --- | --- | --- |
| 1 | FBN1 | 9.55 | Fibrillin-1 |
| 2 | FGG | 8.89 | Fibrinogen gamma chain |
| 3 | FGB | 8.87 | Fibrinogen beta chain |
| 4 | FGA | 8.87 | Fibrinogen alpha chain |
| 5 | FN1 | 8.68 | Fibronectin |
| 6 | DDX17 | 7.63 | DEAD-box helicase 17 |
| 7 | DDX58 | 7.40 | Antiviral innate immune response receptor RIG-I |
| 8 | DDX3X | 7.25 | RNA helicase |
| 9 | FBN2 | 7.09 | Fibrillin 2 |
| 10 | DDX6 | 7.02 | RNA helicase |
| 11 | DDX6 | 7.02 | RNA helicase |
| 12 | LTBP1 | 6.95 | Latent transforming growth factor beta binding protein 1 |
| 13 | EFEMP1 | 6.87 | EGF containing fibulin extracellular matrix protein 1 |
| 14 | MFAP5 | 6.61 | Microfibril-associated protein 5 |
| 15 | NPNT | 6.18 | Nephronectin |
| 16 | VWF | 6.06 | von Willebrand factor (vWF) |
| 17 | DDX5 | 5.74 | Probable ATP-dependent RNA helicase DDX5 |

**Table S4.** Factors and levels of Minitab design

| Level | Code | A | B | C |
| --- | --- | --- | --- | --- |
|  |  | dECM  (w/w, %) | Gelatin concentration  (w/v, %) | Ultrasonic power  (W) |
| High | +1 | 50% | 7.5% | 600 |
| Center | 0 | 37.5% | 6.5% | 400 |
| Low | -1 | 25% | 5.5% | 200 |

**Table S5.** Experiments and results of the factorial design

| Run | Factors | | | Results | |
| --- | --- | --- | --- | --- | --- |
| Order | A | B | C | Pore diameter (μm) | Particle diameter(μm) |
| 1 | 1 | -1 | -1 | 36.34±5.1 | 650.2±15.2 |
| 2 | -1 | -1 | 1 | 40.18±3.3 | 552.7±20.6 |
| 3 | 1 | 1 | 1 | 68.25±13.9 | 532.3±14.5 |
| 4 | -1 | -1 | -1 | 13.57±3.9 | 523.3±11.2 |
| 5 | -1 | 1 | -1 | 25.15±7.4 | 543.7±6.1 |
| 6 | 1 | -1 | 1 | 31.16±11.6 | 484.6±7.5 |
| 7 | -1 | 1 | 1 | 80.09±24.2 | 443.6±8.3 |
| 8 | 1 | 1 | -1 | 28.14±8.3 | 463.1±12.2 |
| 9 | 0 | 0 | 0 | 48.67±10.0 | 690.4±16.3 |
| 10 | 0 | 0 | 0 | 43.20±10.8 | 650.2±6.7 |
| 11 | 0 | 0 | 0 | 54.14±27.5 | 648.7±13.5 |


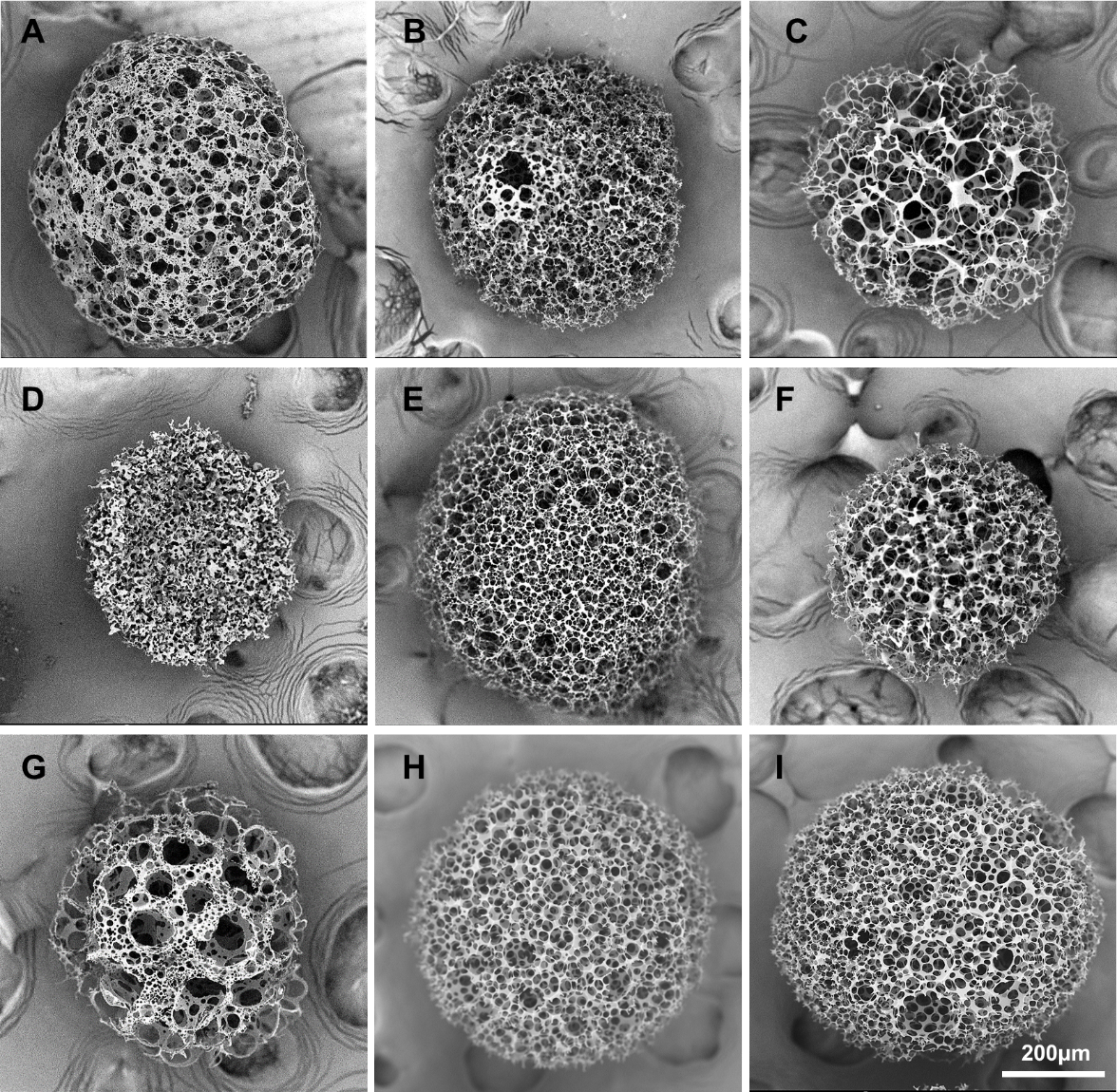


**Figure S1.** SEM images of dECM-PLGA PMs prepared at different experimental conditions based on the Minitab full-factorial design run order as shown in **Table S5** (A: run-1; B: run-2; C: run-3; D: run-4; E: run-5; F: run-6; G: run-7; H: run-8; I: run-9). Scale bar 200 μm.


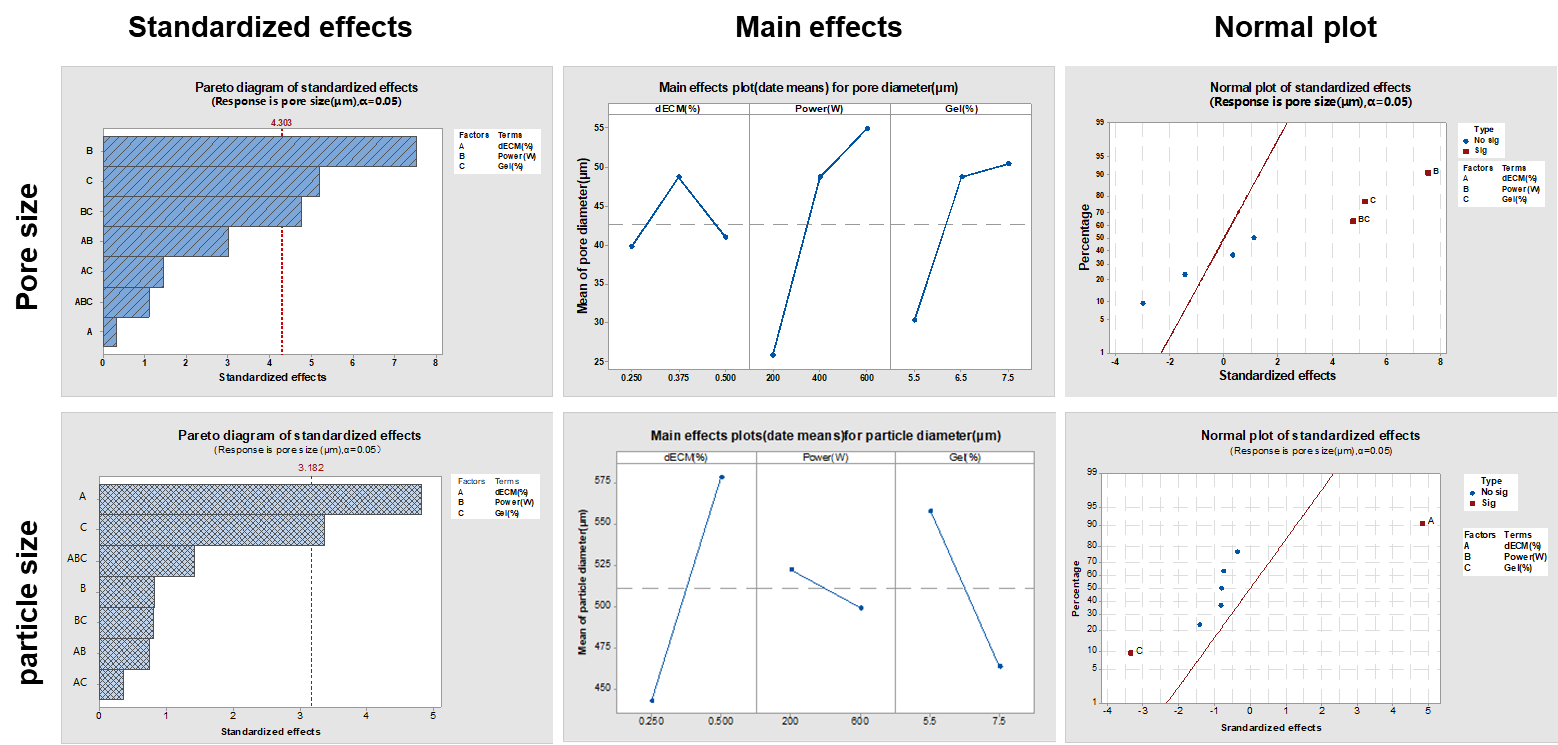


**Figure S2.** Standardized Minitab software design. Standardized effect, main effect, and normal plot of various formulation variables on pore size as well as particle diameters of dECM-PLGA PMs.

**Figure S3.** The pH value variations of PLGA PMs and dECM-PLGA PMs at a condition of constant temperature (37 ℃).


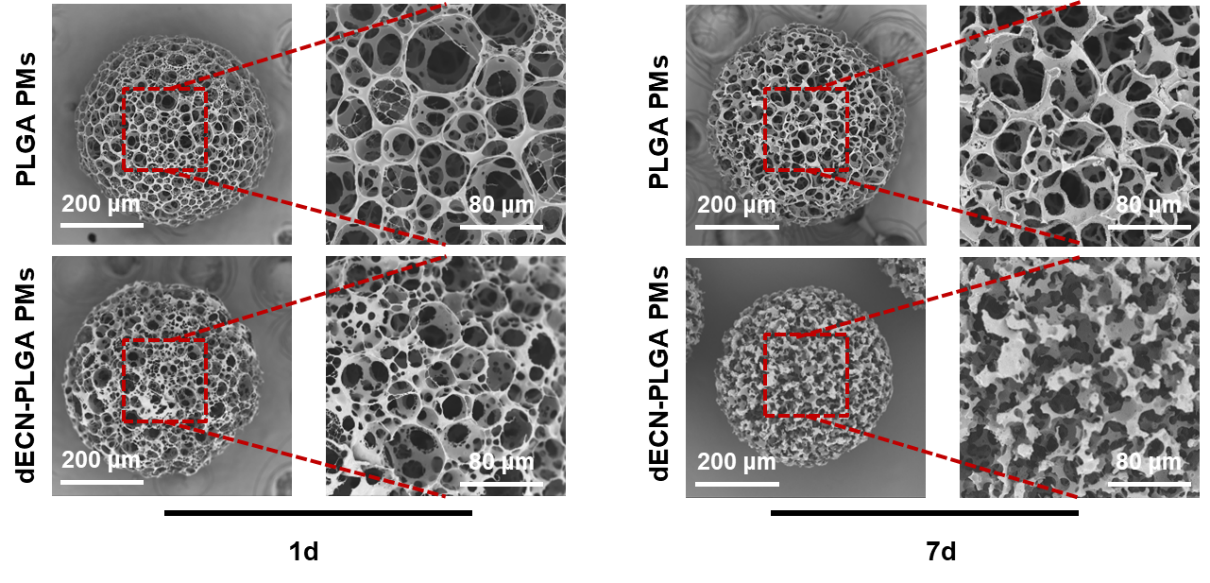


**Figure S4.** SEM morphology analysis for illustrating surface changes of PLGA PMs and dECM-PLGA PMs in PBS (7 d). Scale bar 200 μm (Overview) and 80 μm (Magnified field).

**Figure S5.** Cell viability of L929 after incubation with leach liquor of dECM-PLGA PMs for 48 h at different concentrations.


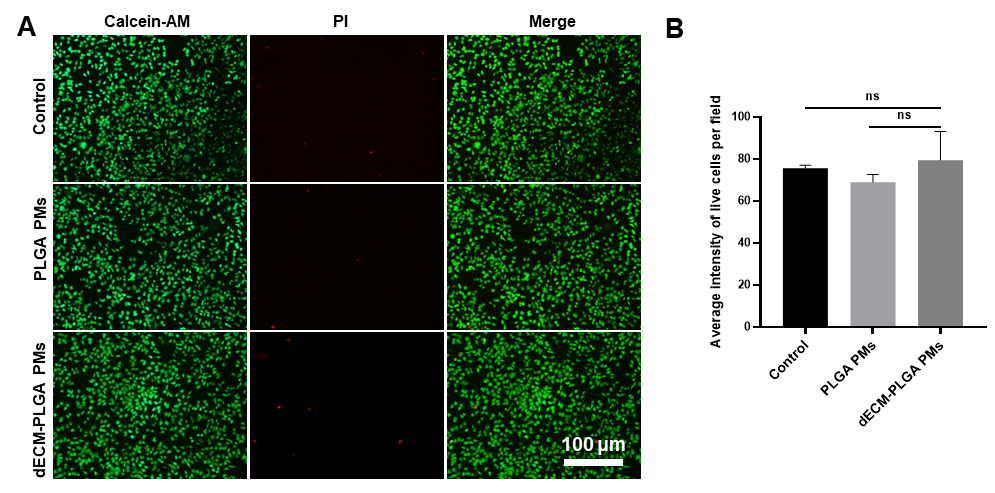


**Figure S6.** (A). Live and dead staining of L929, incubated with leach liquor of dECM-PLGA PMs at different concentrations (48 h). Green = live cells, red = dead cells. Scale bar 100 μm. (B). The average intensity of AM-stained live cells per field of the mouse fibroblast cell line (L929).
